# Comparison of the Functional microRNA Expression in Immune Cell Subsets of Neonates and Adults

**DOI:** 10.3389/fimmu.2016.00615

**Published:** 2016-12-19

**Authors:** Hong-Ren Yu, Te-Yao Hsu, Hsin-Chun Huang, Ho-Chang Kuo, Sung-Chou Li, Kuender D. Yang, Kai-Sheng Hsieh

**Affiliations:** ^1^Department of Pediatrics, Chang Gung Memorial Hospital-Kaohsiung Medical Center, Graduate Institute of Clinical Medical Science, Chang Gung University College of Medicine, Kaohsiung, Taiwan; ^2^Department of Obstetrics, Chang Gung Memorial Hospital-Kaohsiung Medical Center, Graduate Institute of Clinical Medical Science, Chang Gung University College of Medicine, Kaohsiung, Taiwan; ^3^Genomics and Proteomics Core Laboratory, Chang Gung Memorial Hospital-Kaohsiung Medical Center, Graduate Institute of Clinical Medical Science, Chang Gung University College of Medicine, Kaohsiung, Taiwan; ^4^Institute of Biomedical Sciences, Mackay Medical College, New Taipei City, Taiwan; ^5^Department of Pediatrics, Mackay Memorial Hospital, Taipei, Taiwan; ^6^Institute of Clinical Medicine, National Yang Ming University, Taipei, Taiwan

**Keywords:** microRNA, cord blood, leukocyte subsets, let-7b, monocytes

## Abstract

Diversity of biological molecules in newborn and adult immune cells contributes to differences in cell function and atopic properties. Micro RNAs (miRNAs) are reported to involve in the regulation of immune system. Therefore, determining the miRNA expression profile of leukocyte subpopulations is important for understanding immune system regulation. In order to explore the unique miRNA profiling that contribute to altered immune in neonates, we comprehensively analyzed the functional miRNA signatures of eight leukocyte subsets (polymorphonuclear cells, monocytes, CD4^+^ T cells, CD8^+^ T cells, natural killer cells, B cells, plasmacytoid dendritic cells, and myeloid dendritic cells) from both neonatal and adult umbilical cord and peripheral blood samples, respectively. We observed distinct miRNA profiles between adult and neonatal blood leukocyte subsets, including unique miRNA signatures for each cell lineage. Leukocyte miRNA signatures were altered after stimulation. Adult peripheral leukocytes had higher let-7b-5p expression levels compared to neonatal cord leukocytes across multiple subsets, irrespective of stimulation. Transfecting neonatal monocytes with a let-7b-5p mimic resulted in a reduction of LPS-induced interleukin (IL)-6 and TNF-α production, while transfection of a let-7b-5p inhibitor into adult monocytes enhanced IL-6 and TNF-α production. With this functional approach, we provide intact differential miRNA expression profiling of specific immune cell subsets between neonates and adults. These studies serve as a basis to further understand the altered immune response observed in neonates and advance the development of therapeutic strategies.

## Introduction

Differences in the expression of biological molecules in the immune cells of newborns and adults contribute to diverse cell function and atopic properties (1–5). These immune differences are reflected in varied immune responses, cellular subset composition, cytokine production, and cellular/humoral protein levels (3, 4, 6, 7). Other mediators including interleukin (IL)-10, prostaglandin E2, and progesterone produced by the placenta can also upregulate Th2 differentiation, resulting in downregulation of Th1 responses (8–10). In the previous studies, we found that neonates have selectively impaired IFNα-mediated Th1 immune responses (3, 11). By using proteomic tools, we identified at least 34 differentially expressed proteins between adult peripheral blood mononuclear cells (PBMCs) and cord blood mononuclear cells (CBMCs). There were also validated cytoskeletal differences between PBMCs and CBMCs (12). Moreover, we observed a decrease in adenosine deaminase and an increase in arginase-1 in neonatal mononuclear cells (MNCs), which was associated with impaired immune function (6). Different modulatory effects of adenosine and l-arginine on neonatal and adult leukocytes have also been investigated (4).

microRNAs (miRNAs) are small (19–22 nt) single-stranded non-coding RNA molecules derived from hairpin-structured precursors (13). These miRNAs function by directly binding to the indicated 3′-untranslated region of specific target mRNA, leading to target mRNA degradation or translational repression. miRNAs have been shown to play important roles in human development, cellular differentiation and homeostasis, adaptation, oncogenesis, and host cell interactions with pathogens (14–16). miRNAs are also involved in the regulation of immune systems, indicating that they modulate many aspects of the immune response, such as differentiation, proliferation, and activation of intracellular signaling pathways (17–19).

Recently, the essential regulatory roles of specific miRNAs in neonatal immune responses have also been noted. miR-184 was reported to regulate NFAT1 in neonatal CD4 T cells (20), while miR-146a and miR-155 downregulated toll-like receptor (TLR) signals in neonatal monocytes and plasmacytoid dendritic cells (pDCs), respectively (21, 22). We also found that miR-125b negatively regulates LPS-induced TNF-α expression in neonatal monocytes (23). It is possible that there is greater diversity in miRNA expression in neonatal leukocytes, which may contribute to the unique immunity of neonates.

Accumulating evidence demonstrates that miRNAs show specific signatures in different blood cell lineages and various stages of cellular differentiation (24–26). Since different cell types have unique functions and correspondingly, distinct gene expression profiles, determining the specificity of miRNA expression profiles in different leukocyte subpopulations is very important for both understanding the biology of the immune system and for characterization. Our hypothesis is that distinct miRNA profiles of different leukocyte subpopulations from neonatal and adult samples contribute to their relatively different immune responses. To exhibit proper immune functions, the immune cells must undergo activation, proliferation, and cytokine production upon encountering antigens (27). The miRNA profiles of leukocytes from neonates and adults have been widely reported. However, an important limitation of these studies was that they did not directly compare activated leukocytes of cord blood (CB) with those from adult peripheral blood, but instead used resting leukocytes. This approach is not as informative for identifying true differences in the functional transcriptome. In other studies, total leukocytes were investigated rather than unique leukocyte subsets (28). This approach is also information limiting because leukocyte subsets have distinct functions. In this study, we set out to comprehensively analyze the miRNA expression signatures of eight leukocyte subsets [polymorphonuclear cells (PMNs), monocytes, CD4^+^ T cells, CD8^+^ T cells natural killer (NK) cells, B cells, pDCs, and myeloid dendritic cells (mDCs)] between neonatal and adult samples. The miRNA profile of activated and resting leukocytes was also analyzed. With this functional approach, we provide differential miRNA expression profiling of specific immune cell subsets between neonates and adults. This provides a basis for further understanding of the altered immune response in neonates and can guide the development of novel therapeutic strategies.

## Materials and Methods

### Collection of Human Umbilical Cord Blood and Adult Peripheral Blood and Cell Separation

Human umbilical CB was collected in heparinized tubes (10 U/ml) by cordocentesis at the time of elective Cesarean section or normal spontaneous delivery of healthy mothers, following the receipt of informed consent. About 20–50 ml of CB was obtained from each case. The peripheral blood samples were obtained from healthy adult volunteers aged 20–40 years. Approximately 50–100 ml of blood was obtained from each healthy adult depending on the experimental design. Heparinized blood samples were collected, and the plasma was stored at −80°C before analysis. The leukocyte separation protocol was utilized as previously described (11, 12). Briefly, whole blood was mixed with 4.5% (w/v) dextran (Amersham Pharmacia Biotech, Uppsala, Sweden) sedimentation at a ratio of 1:5 to separate leukocytes from red blood cells (RBCs) for 30 min. Leukocytes were then separated into PMNs and MNCs by density gradient centrifugation in Ficoll-Plaque™ (Amersham Pharmacia) at a ratio of 2:1 at 1,500 rpm for 30 min at 20°C. After centrifugation over a Ficoll cushion, MNCs were washed and counted on a hemocytometer by trypan blue staining. The PMN fraction in MNCs was less than 1% in adult and neonate samples. The study protocol was approved by the institutional review board of the study hospital.

### Cell Separation and Enrichment

CD4^+^ T-cells, CD8^+^ T-cells, CD14^+^ monocytes, or CD56^+^ NK cells were separated from MNCs using the IMag Cell Separation Systems (BD Biosciences, San Jose, CA, USA) following the protocol supplied by the manufacturer. In brief, per 2 × 10^7^ of MNCs pellet was suspended in 100 µl of anti-human CD4, CD8, CD14, or CD56 magnetic particles (BD Biosciences) and incubated at room temperature for 30 min. Then, the labeled cells were resuspended in 1× BD IMag™ buffer, and the tubes were placed in the BD Imagnet™ (BD Biosciences) for 10 min. With the tube in the BD IMagnet™, the supernatant was removed to discard the undesired leukocytes. To maximize purity, the process was repeated three times. Then, the indicated cell fraction was carefully washed and suspended in PBS or medium. For pDCs isolation, dead cells were initially depleted by magnetic negative selection with Dead Cell Removal Kit (Miltenyi. Biotec, Bergisch Gladbach, Germany), then per 5 × 10^7^ of MNCs pellet was isolated with 50 µl of CD304 (BDCA-4)-microbeads (Miltenyi Biotech) and magnetic columns according to the manufacturer’s instructions. The mDCs were isolated by magnetic positive selection with CD1c (BDCA-1)-microbeads and a magnetic column (Miltenyi Biotech) following the depletion of CD19^+^ cells. The purity of isolated cells was confirmed by flow cytometry, and all isolated cells demonstrated greater than 90% purity (12).

### Cell Culture and Stimulation

Approximately 1 ml of leukocytes (2 × 10^6^ cells/ml) suspended in RPMI-1640 medium containing 10% heat-inactivated fetal bovine serum were co-cultured with and without the indicated stimulatory agents in 24-well culture plates. The cells were incubated in a humidified atmosphere of 5% CO_2_ at 37°C. PMNs, monocytes, and mDCs were stimulated with LPS (100 ng/ml) for 6 h and CD4^+^ and CD8^+^ T cells, B-cells, and NK cells were stimulated with PHA (5 µg/ml) for 24 h. The stimulation condition for pDCs was CpG-ODN 2,216 (5 µg/ml) for 16 h as previously reported (29). Cells were harvested after culture and subjected to miRNA expression analysis.

### RNA Isolation

Total RNA was extracted using the TRIzol^®^ Reagent (Invitrogen, USA) according to the manufacturer’s instructions. Purified RNA was quantified at OD260 nm using a ND-1000 spectrophotometer (Nanodrop Technology, USA) and qualitatively analyzed using a Bioanalyzer 2100 (Agilent Technology, USA) with RNA 6000 nano lab-chip kit (Agilent Technologies, USA).

### RNA Labeling and Hybridization

Total RNA (0.1 µg) was dephosphorylated and labeled with pCp-Cy3 using the Agilent miRNA Complete Labeling and Hyb Kit (Agilent Technologies, USA, microRNA Spike-In Apply). Hybridization buffer (2×) (Agilent Technologies, USA) was added to the labeled mixture at a final volume of 45 µl. The mixture was heated for 5 min at 100°C and immediately cooled to 0°C. Each 45 µl sample was hybridized onto an Agilent human miRNA Microarray R19 (Agilent Technologies, USA) at 55°C for 20 h. After hybridization, slides were washed for 5 min in Gene Expression Wash Buffer 1 at room temperature and then washed for 5 min in Gene Expression Wash Buffer 2 at 37°C. Microarrays were scanned using the Agilent microarray scanner (Agilent Technologies, model G2505C) at 535 nm for Cy3. Feature Extraction (Agilent Technologies) software version 10.7.3.1 was used for image analysis. If both channels produced intensities less than 100 for a given miRNA, that spot was filtered out. For spots with one channel intensity less than 100 and the other 100 or greater, the signal that was less than 100 was set to 100 prior to calculation of the signal ratio. Hierarchical clustering was performed using the Genesis software (Graz University of Technology), and Pearson’s correlation was calculated as the distance metric.

### miRNA Array

In this study, we used Agilent SurePrint Human miRNA Microarray chips (Release 19.0, Agilent Technologies, Santa Clara, CA, USA) to globally evaluate the miRNA expression profiles of the samples. The extracted RNA samples were first subjected to quality examination with a Bioanalyzer to check whether they passed the criterion, RIN ≥ 7.0. Then, 0.5 µg of the qualified RNA samples were prepared using the Agilent SurePrint Human miRNA Microarray kits, followed by microarray chip assays. The generated microarray data were analyzed with Partek (Partek Inc., St. Louis, MO, USA) under quantized normalization and log 2 transformation. Total 32 miRNA array chips were used for 8 kinds of leukocytes with/without indicated stimulation from CB and adult peripheral blood.

### Quantitative Reverse Transcription PCR (qRT-PCR) Analysis of miRNAs

Total RNA was extracted from the cell pallet using TRIzol^®^ reagent (Invitrogen, Carlsbad, CA, USA) according to the manufacturer’s protocol. qRT-PCR analysis was performed based on two-step qRT-PCR (30). Briefly, in the reverse transcription (RT) step, each RNA sample was reverse transcribed to cDNA using the TaqMan^®^ miRNA Reverse Transcription Kit (#PN 4366597, Applied Biosystems, Foster City, CA, USA). Each reaction contained 10 ng of RNA, 50 U of Multiscribe Reverse Transcriptase, 100 mM of deoxyribonucleotide triphosphate, 1× reaction buffer, 4U of RNase inhibitor, and 1× specific miRNA primers (TaqMan MicroRNA Assay, PN 4427975, Applied Biosystems) with nuclease-free H_2_O to 15 µl of final volume. The reaction mixture was then incubated at 16°C for 30 min, followed by incubation at 42°C for 30 min. The enzyme was then inactivated at 85°C for 5 min. In the TaqMan real-time PCR step, 2 µl of cDNA solution was amplified using 1× specific miRNA primers (TaqMan MicroRNA Assay), 1× TaqMan Universal Master Mix II (#PN 44440040, Applied Biosystems, Darmstadt, Germany), and nuclease-free H_2_O to 10 µl of final volume. Quantitative PCR was performed on a 7500 System quantitative PCR machine (Applied Biosystems, Darmstadt, Germany) using a two-step PCR protocol with a denaturation step at 95°C for 10 min, followed by 40 cycles with a denaturation step at 95°C for 15 s, and an annealing/elongation step at 60°C for 60 s. The cycle threshold (Ct) values were calculated using the SDS 2.1 software (Applied Biosystems, Darmstadt, Germany). The relative amount of miRNA to small nuclear U6 snRNA (the internal control) was calculated using the equation 2^–∆CT^, where ΔCt = (Ct microRNA—Ct U6RNA) data were analyzed as previously described (31). The target miRNA primer sequences were as follows: hsa-let-7b-5p (002619): 5′-UGA GGU AGU AGG UUG UGU GGU U-3′; hsa-miR-29a-3p (002112): 5′-UAG CAC CAU CUG AAA UCG GUU A-3′; hsa-miR-29b-3p (000413): 5′-UAG CAC CAU UUG AAA UCA GUG UU-3′; hsa-miR-130a-3p (000454), 5′-CAG UGC AAU GUU AAA AGG GCA U-3′. hsa-U6 snRNA (001973).

### Transfection of let-7b-5p Mimic and Inhibitor with Functional Validation

A total of 2.5 × 10^5^ cells/0.5 ml primary human CD14^+^ monocytes were maintained in RPMI-1640 medium supplemented with 10% FBS, 100 U/ml penicillin, 100 µg/ml streptomycin, and 0.25 µg/ml Amphotericin B (GIBCO) at 37°C in a humidified incubator containing 5% CO_2_. For functional analysis, Has-let-7b-5p mimics (#47-775, EXIQON, TGAGGTAGTAGGTTGTGTGGTT), Has-let-7b-5p miRCURY LNA™ microRNA inhibitor (#4100945, EXIQON, ACCACACAACCTACTACCTC), cel-miR-39-3p miRCURY LNA™ microRNA Mimic Negative Control (#479902, EXIQON, TCACCGGGTGTAAATCAGCTTG), and miRCURY LNA™ microRNA inhibitor scramble control (#199900, EXIQON, ACGTCTATACGCCCA) were used. The miR mimics and LNA inhibitors were transfected into CD14^+^ cells using TransIT-X2 (Mirus, Madison, WI, USA) according to the manufacturer’s instructions. Briefly, cells were plated in a 48-well plate. miR mimics (50 µl) or LNA inhibitors and 1.5 µl of TransIT-X2 Transfection Reagent were added separately to 450 µl of RPMI-1640 medium (Invitrogen). The solution was then mixed to form the transfection complex. The transfection complex was added to cells with a final working concentration 20 or 40 nM and incubated at 37°C for 24 or 48 h, as indicated. After incubation with the transfection complex, the transfected cells were stimulated with or without LPS (100 ng/ml) for 16 h. The culture supernatants were then collected for quantification of IL-6, IL-8, and TNF-α production by ELISA (R&D Systems, Minneapolis, MN, USA, catalog number DY260,208 and DY210).

### Statistics

Data are expressed as the mean ± SEM. The Mann–Whitney *U* test was used when two groups were analyzed. Results with a *p*-value of less than 0.05 were considered to be statistically significant. All statistical tests were performed using the SPSS 19.0 for Windows XP (SPSS, Inc., Chicago, IL, USA).

## Results

### Hierarchical Clustering of Adult Peripheral Blood and Cord Blood Leukocyte Subpopulations Based on miRNA Expression

**Figure 1:**
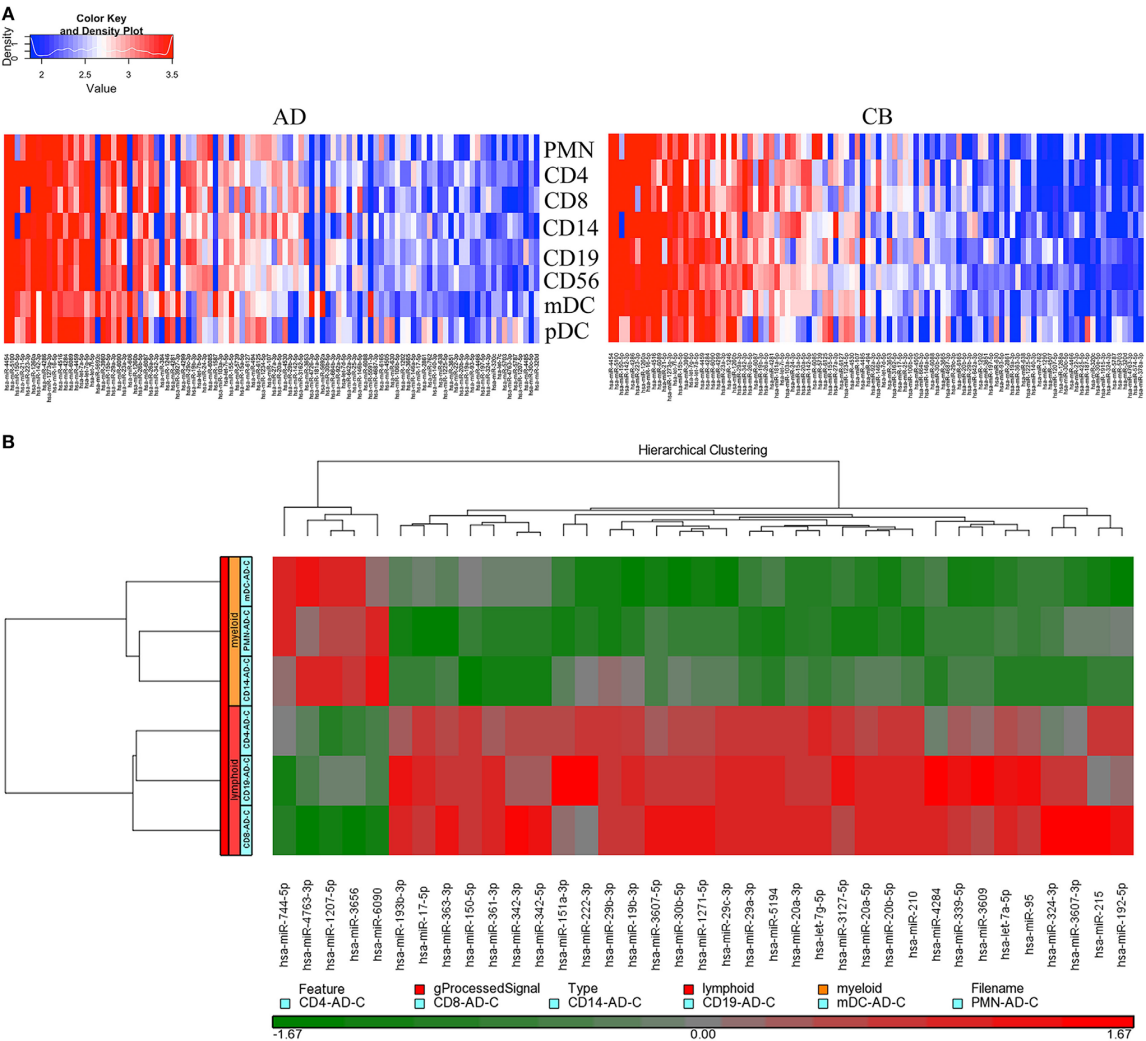
**Distinct microRNA (miRNA) expression between adult and neonatal leukocytes**. **(A)** Neonate leukocyte subsets show distinct miRNA expression compared to adult leukocytes. Heat map showing distinct expression of miRNAs from different leukocyte subsets of adult peripheral blood (left) and cord blood (right). Each heat map lane represents the top 100 expressed miRNAs in each leukocyte subset. Higher intensity is shown in red and lower intensity in blue. **(B)** Myeloid and lymphoid leukocytes have unique miRNA profiles. Heat map showing distinct expression of miRNAs from myeloid (polymorphonuclear cells, CD14^+^ monocytes, and myeloid dendritic cells) and lymphoid (CD4^+^ T cells, CD8^+^ T cells, and CD19^+^ B-cells) leukocyte subsets. Thirty-seven miRNAs with the highest transcript levels across all tested cell lineages were shown. Higher intensity is shown in red and lower intensity in green.

According to the manufacturer’s design, probe sets displaying a log 2 signal value greater than 6 were linear and therefore reliable for miRNA analysis. Using data from the pooled samples, specific probe sets displayed an average log 2 signal value greater than 6 in at least one cell type and were deemed to be expressed above background. PMNs, CD4^+^ T cells, CD8^+^ T cells, CD14^+^ monocytes, and CD56^+^ NK cells were isolated from healthy adult peripheral or CB obtained from pools of five donors each. The BDCA1^+^ mDCs were obtained from pools of 10 healthy adult peripheral or CB donors. The BDCA4^+^ pDCs were obtained from pools of 19 healthy adult peripheral blood and 31 CB donors for its little amount. Subjects were randomized across pools for gender. We chose to pool samples in order to get sufficient cell numbers for rare cell types and to decrease sample variation. Because of the rare amount of dendritic cells, normalized miRNA expression data from one chip array with pooled leukocytes were analyzed in this study. The array data have been submitted to GEO and assigned an *accession number* “*GSE89853*.” The raw data are now available and open for the researcher community. Full lists of normalized miRNA expression are shown in Table S1 in Supplementary Material. An unsupervised hierarchical cluster analysis of the top 100 most abundant miRNAs revealed discrete miRNA profiles for adult and CB leukocyte subsets, including unique miRNA signatures for each cell lineage (Figure 1A).

In order to characterize the specific miRNA expression between myeloid and lymphoid leukocytes, adult leukocyte subsets were divided into myeloid (PMNs, monocytes, and mDCs) and lymphoid groups (CD4^+^, CD8^+^ T cells, and CD19^+^ B-cells). The miRNA profiles of these two groups were compared as shown in Figure 1B. Samples from the same origin (myeloid or lymphoid) had similar miRNA expression profiles and were clustered together. Myeloid and lymphoid leukocytes have unique miRNA profiles that demonstrate the difference in their development. miR-744-5p, miR-4763-3p, miR-1207-5p, miR-3656, and miR-6090 were predominantly expressed in myeloid cells. In contrast, let-7g-5p (especially in CD4^+^ T cells), miR-342-3p, miR-324-3p, miR-3607-3p, miR-215 (especially in CD8^+^ T cells), let-7a-5p, miR-95, miR-151a-3p, miR-222-3p, miR-3127-5p, miR-4284, and miR-3609 (especially in B-cells) were expressed mainly in lymphoid cells. Interestingly, two members of the let-7 family (let-7a-5p and let-7g-5p) were present in the set of abundantly expressed miRNAs in lymphoid cells.

### miRNA Signature Distinguished Adult and Neonatal Leukocyte Subsets

**Figure 2:**
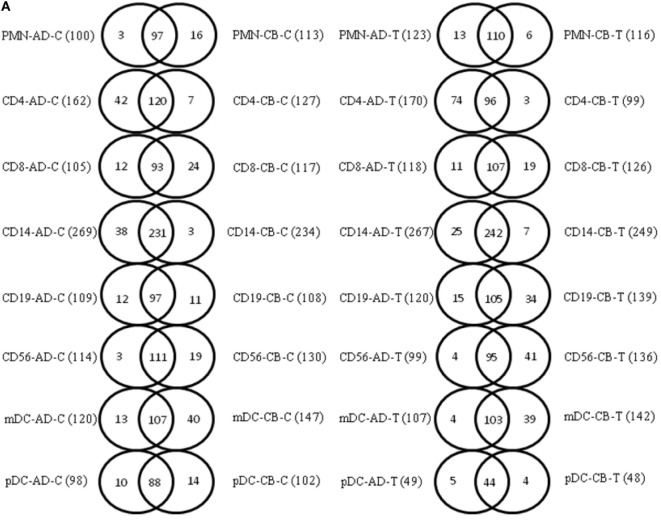

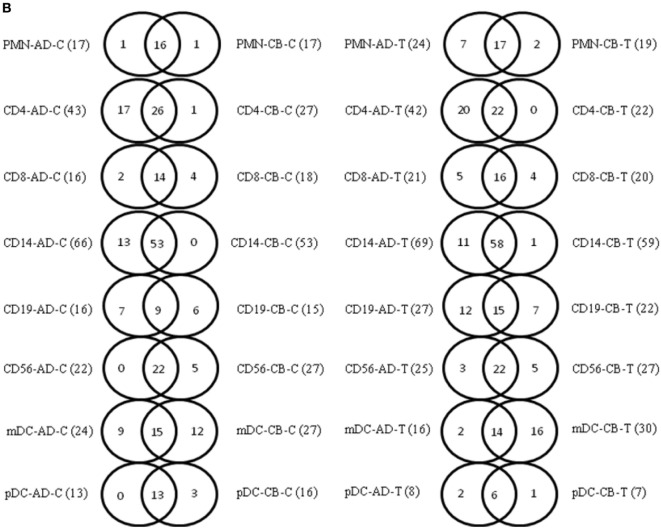
**Common and unique microRNA (miRNA) transcripts exist in adult and neonatal leukocyte subsets**. **(A)** Venn diagrams showing comparative analysis of common and unique miRNA transcripts in the corresponding adult and cord blood (CB) leukocyte subsets (array intensity log 2 greater than 6). **(B)** Venn diagrams showing comparative analysis of common and unique miRNA transcripts in the corresponding adult and CB leukocyte subsets (array intensity log 2 greater than 10). Abbreviations: AD-C, adult blood cells without stimulation; CB-C, cord blood cells without stimulation; AD-T, adult blood cells with stimulation; CB-T, cord blood cells with stimulation.

A lower filter threshold (log 2 signal value greater than 6) was initially set for miRNA analysis. Adult CD14^+^ monocytes demonstrated higher miRNA expression (269 miRNAs) than other leukocyte subsets, while adult pDCs had the lowest miRNA expression (98 miRNAs) (Table S2 in Supplementary Material). When comparing adult and CB leukocyte subsets, the Venn diagrams also showed common and unique miRNA transcripts in indicated leukocytes before and after stimulation (Figure 2). There were significant overlaps between adult and neonatal leukocyte subsets. We also applied a higher filter (log 2 signal value greater than 10) to identify the highly expressed miRNA transcripts among the different leukocyte subsets. With the higher filter, adult CD14^+^ monocytes had the highest number of miRNAs (66 miRNAs) compared to other leukocyte subsets, while adult pDCs had the lowest number of miRNAs (13 miRNAs) (Table S2 in Supplementary Material). Figure 2B demonstrates the common and unique miRNA transcripts in indicated leukocytes subsets between adult and CB with the higher filter. Significant overlaps between the adult and neonatal leukocyte subsets were also observed with the higher filter range. Using a higher filter, mDCs had the least overlap of miRNAs between adult and neonatal leukocytes (5/26; 19.2% overlap). These common and unique miRNA transcripts detail between indicated adult and CB leukocyte subsets in Table S3 in Supplementary Material.

### Leukocytes miRNA Signature Altered after Stimulation

**Figure 3:**
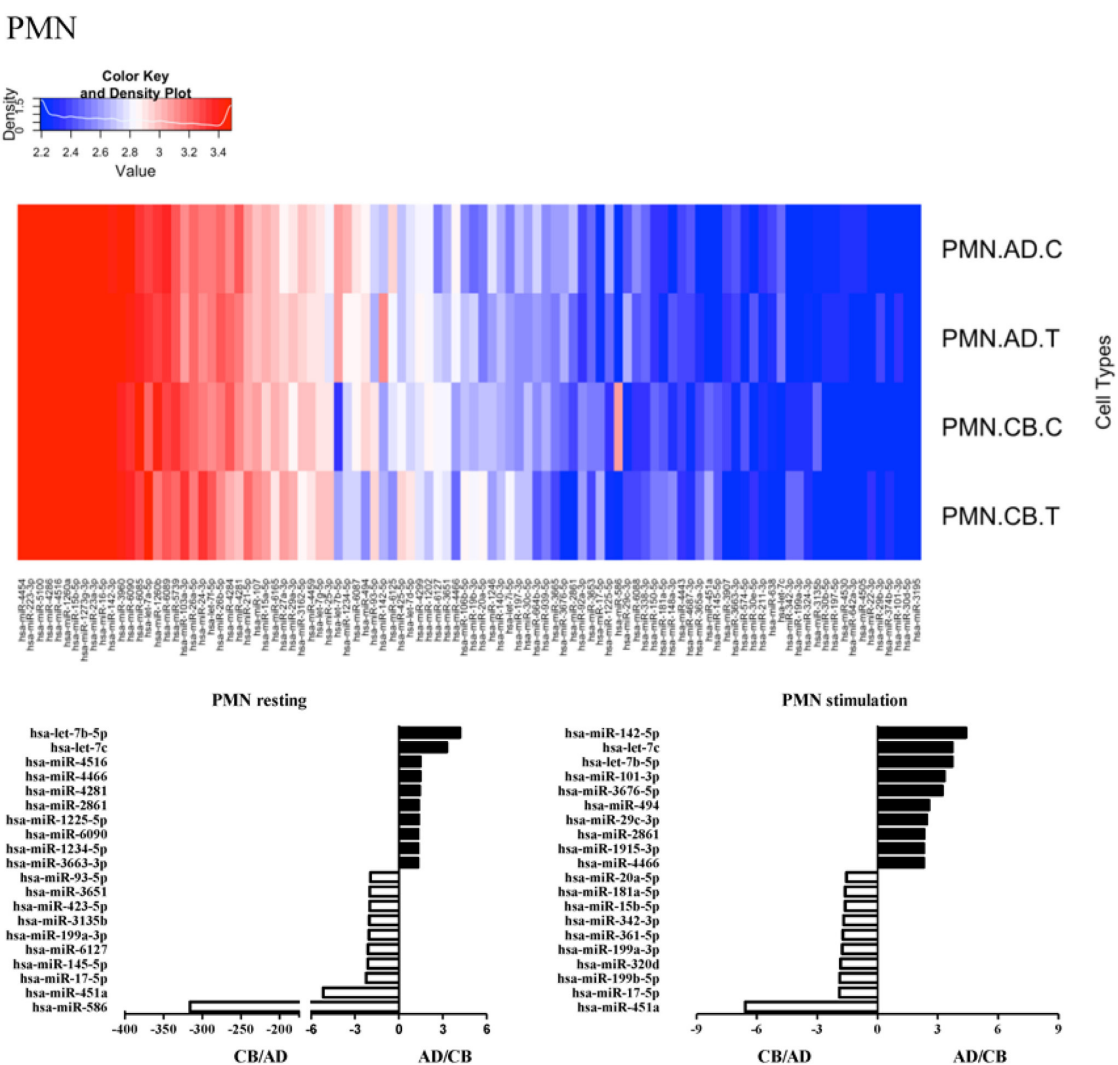
**Differential microRNA (miRNA) profiles between adult and neonatal polymorphonuclear cells (PMNs)**. Upper heat map showing distinct miRNA expression in activated and resting leukocyte subsets of adult peripheral blood and cord blood. Each heat map lane represents the top 100 expressed miRNAs in each leukocyte subset. Higher intensity is shown in red and lower intensity in blue. Lower bar charts showing the top 10 differentially expressed miRNAs in adult (black bar) or (white bar) neonate cells in specific leukocytes at resting (left) or activated states (right). The expression of miRNAs is represented as relative folds. Has, *Homo sapiens*.

**Figure 4:**
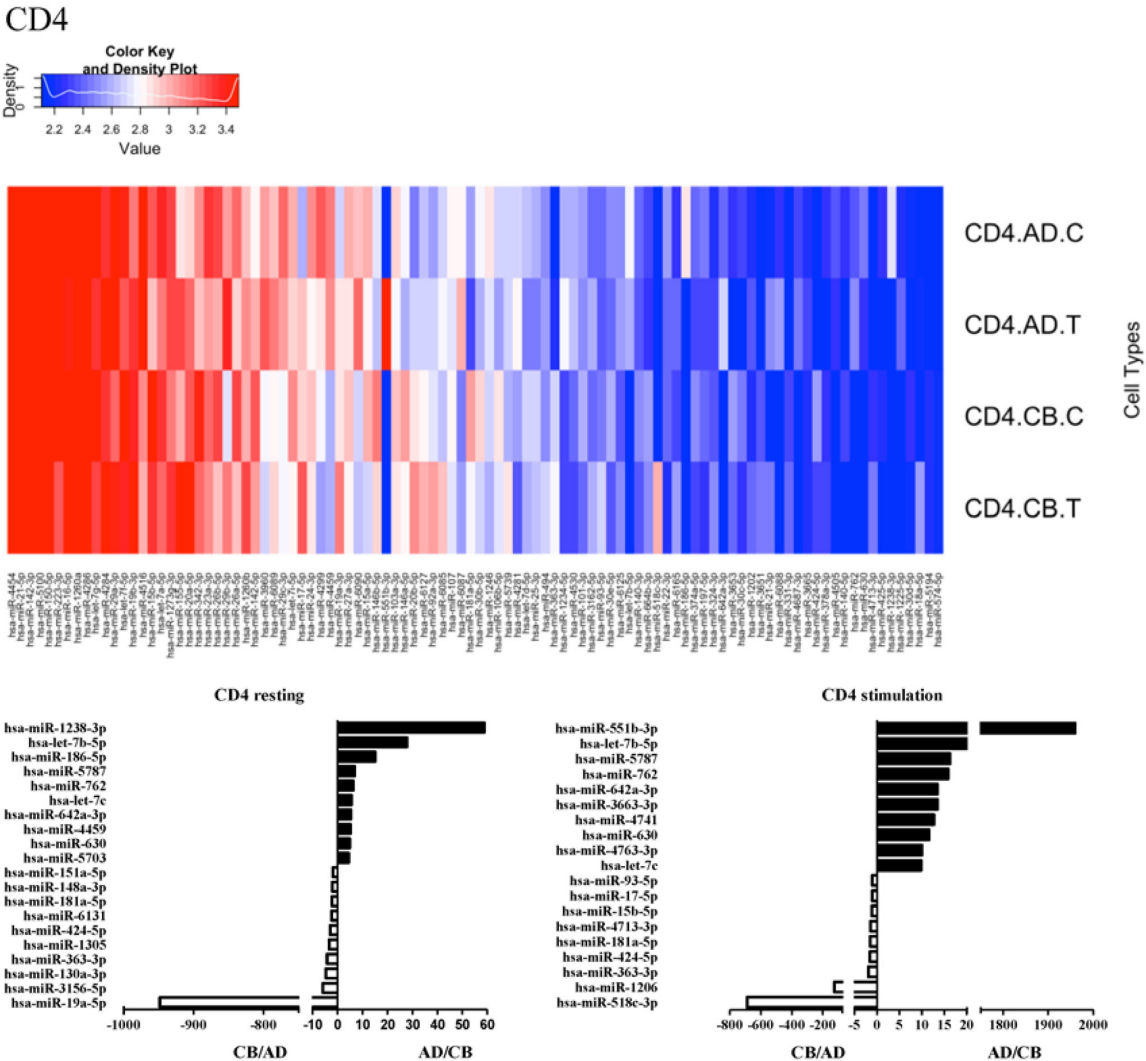
**Differential microRNA profiles between adult and neonatal CD4^+^ T cells**.

**Figure 5:**
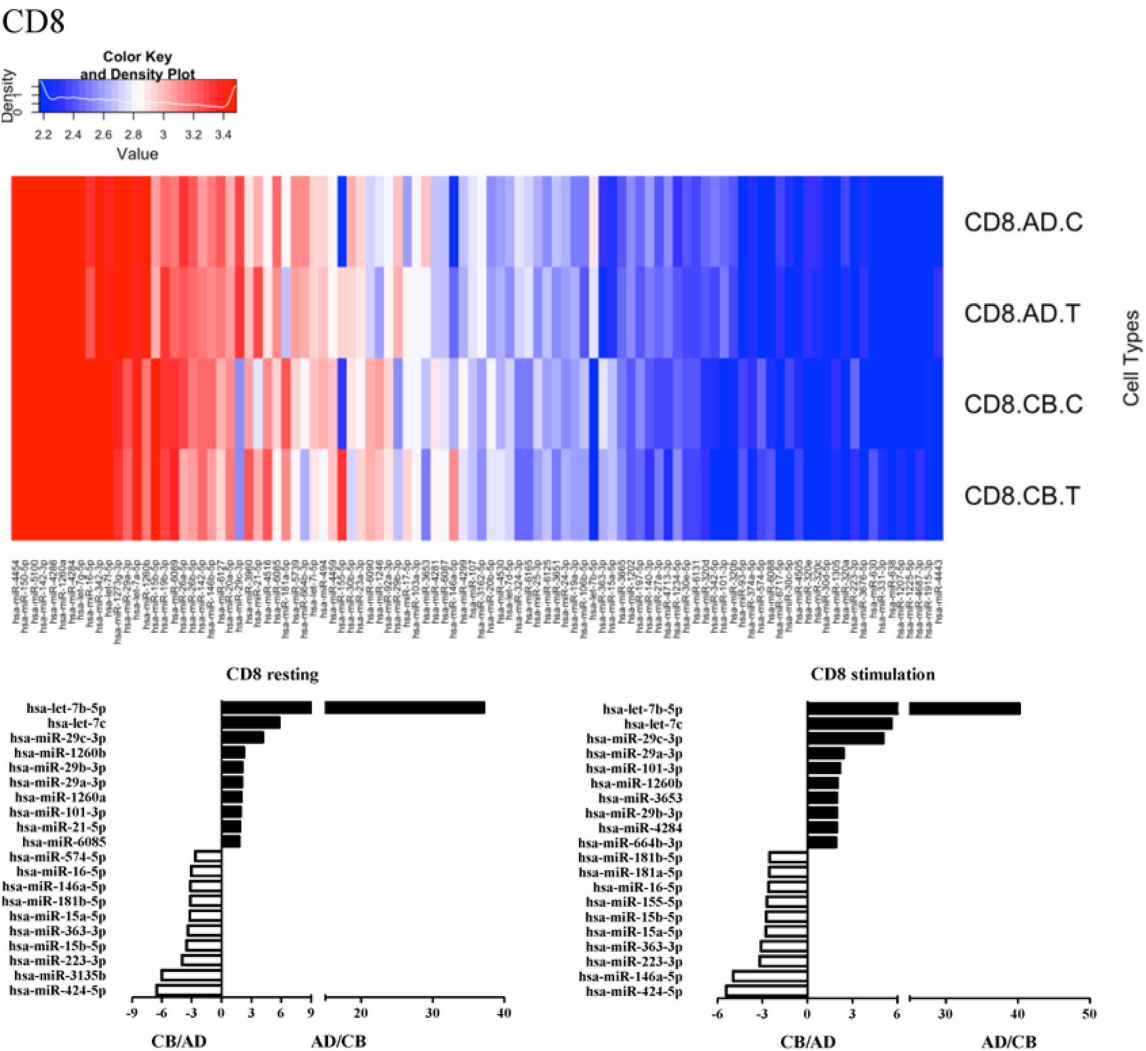
**Differential microRNA profiles between adult and neonatal CD8^+^ T cells**.

**Figure 6:**
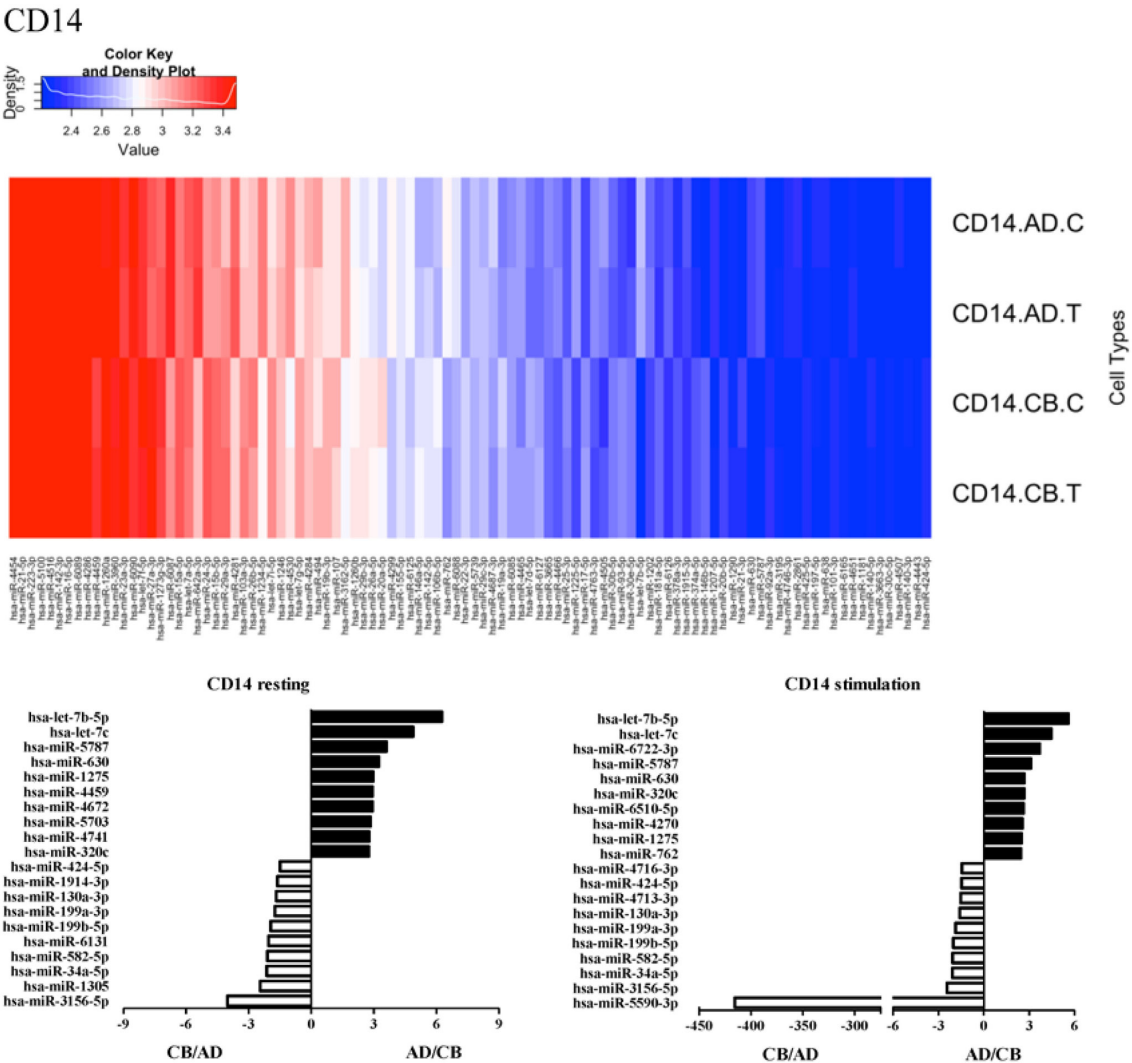
**Differential microRNA profiles between adult and neonatal CD14^+^ monocytes**.

**Figure 7:**
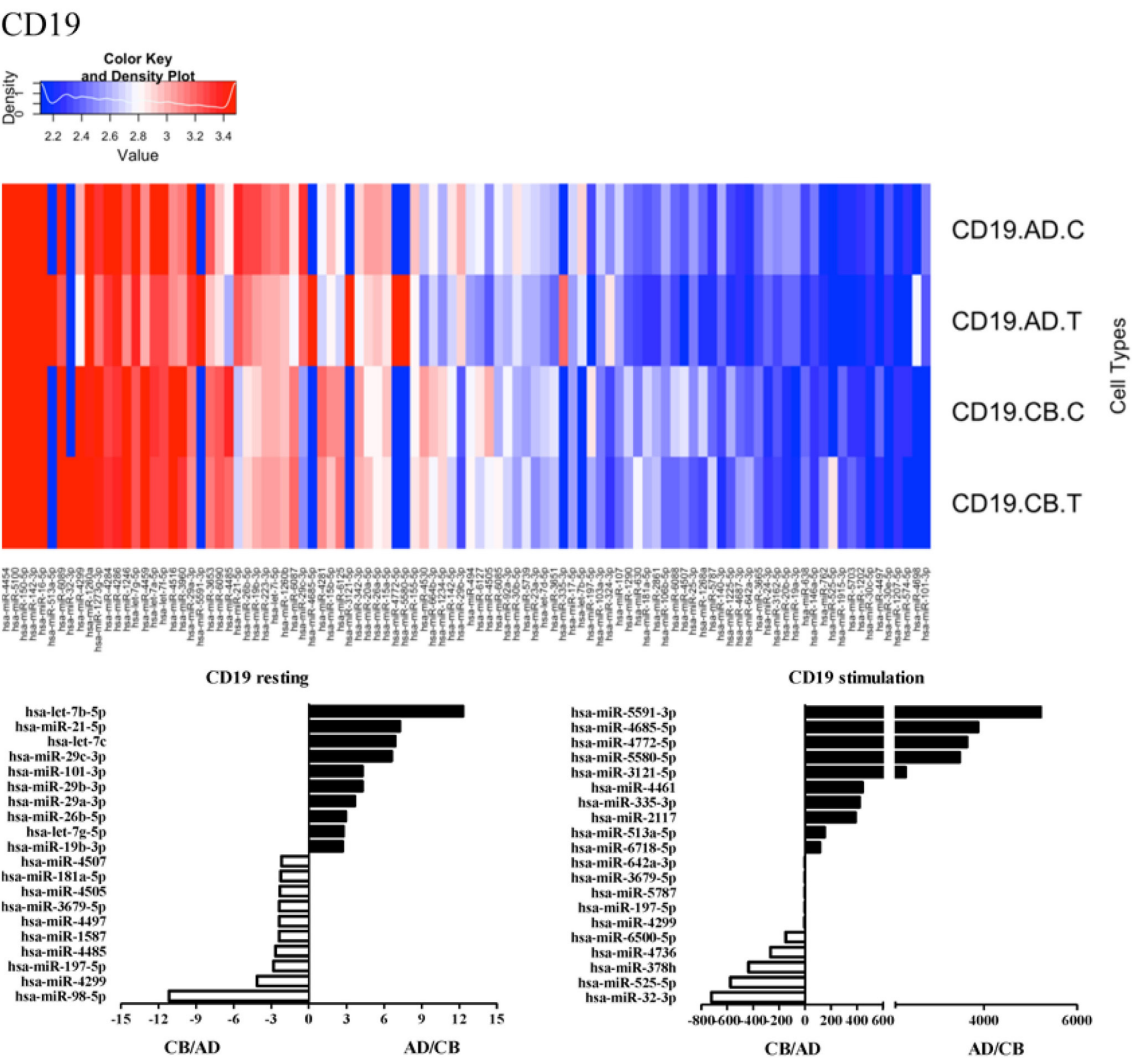
**Differential microRNA profiles between adult and neonatal CD19^+^ B-cells**.

**Figure 8:**
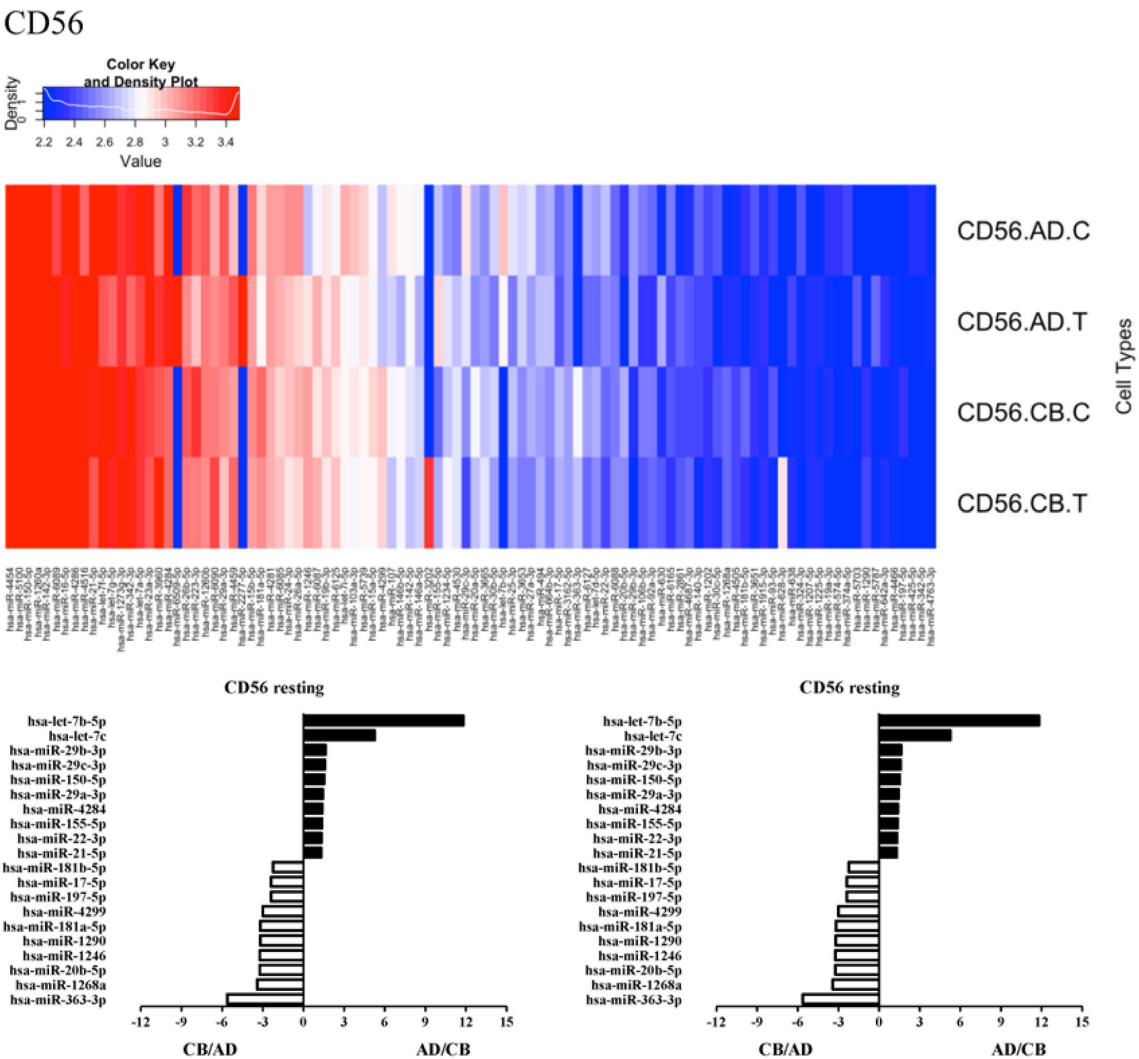
**Differential microRNA profiles between adult and neonatal CD56^+^ natural killer cells**.

**Figure 9:**
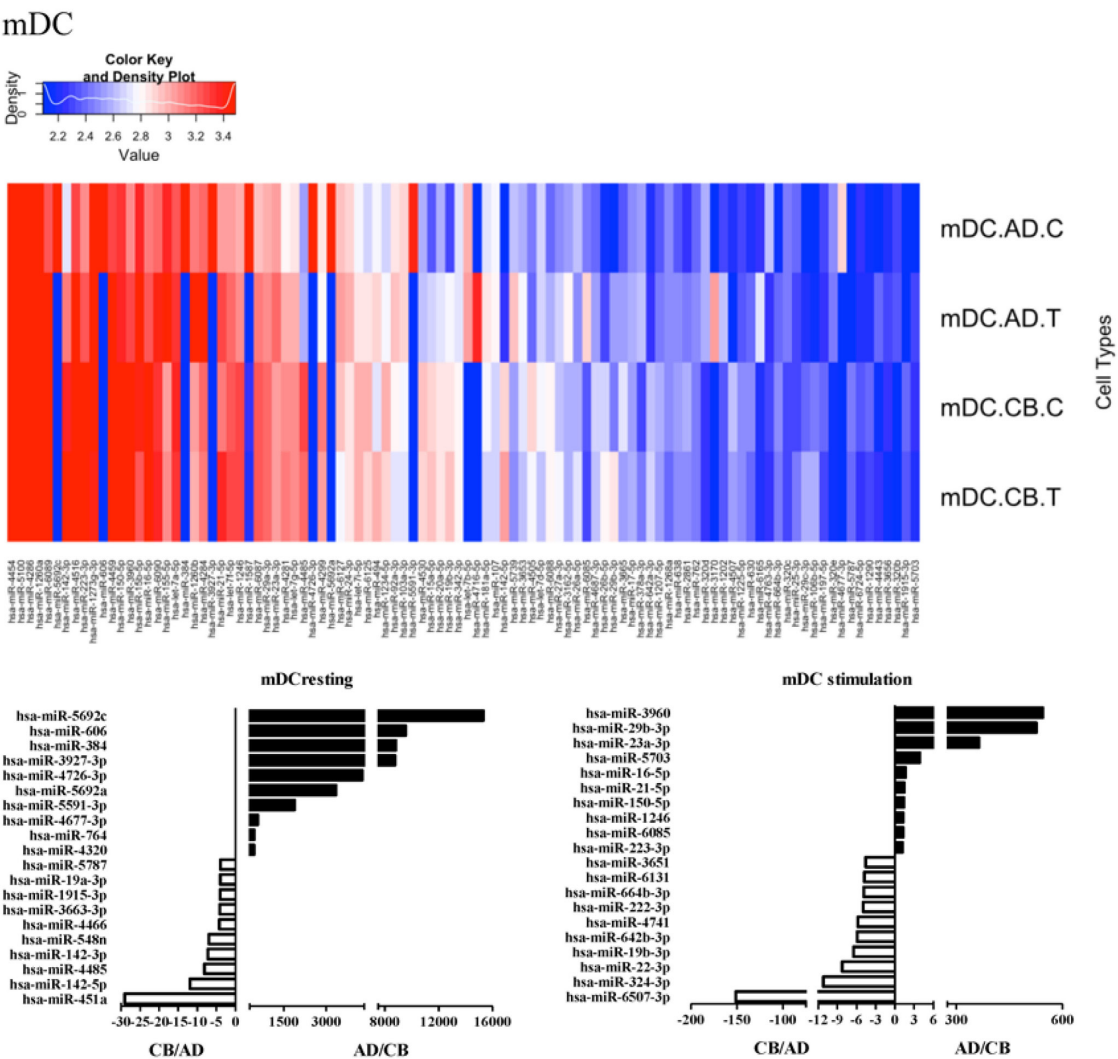
**Differential microRNA profiles between adult and neonatal myeloid dendritic cells**.

**Figure 10:**
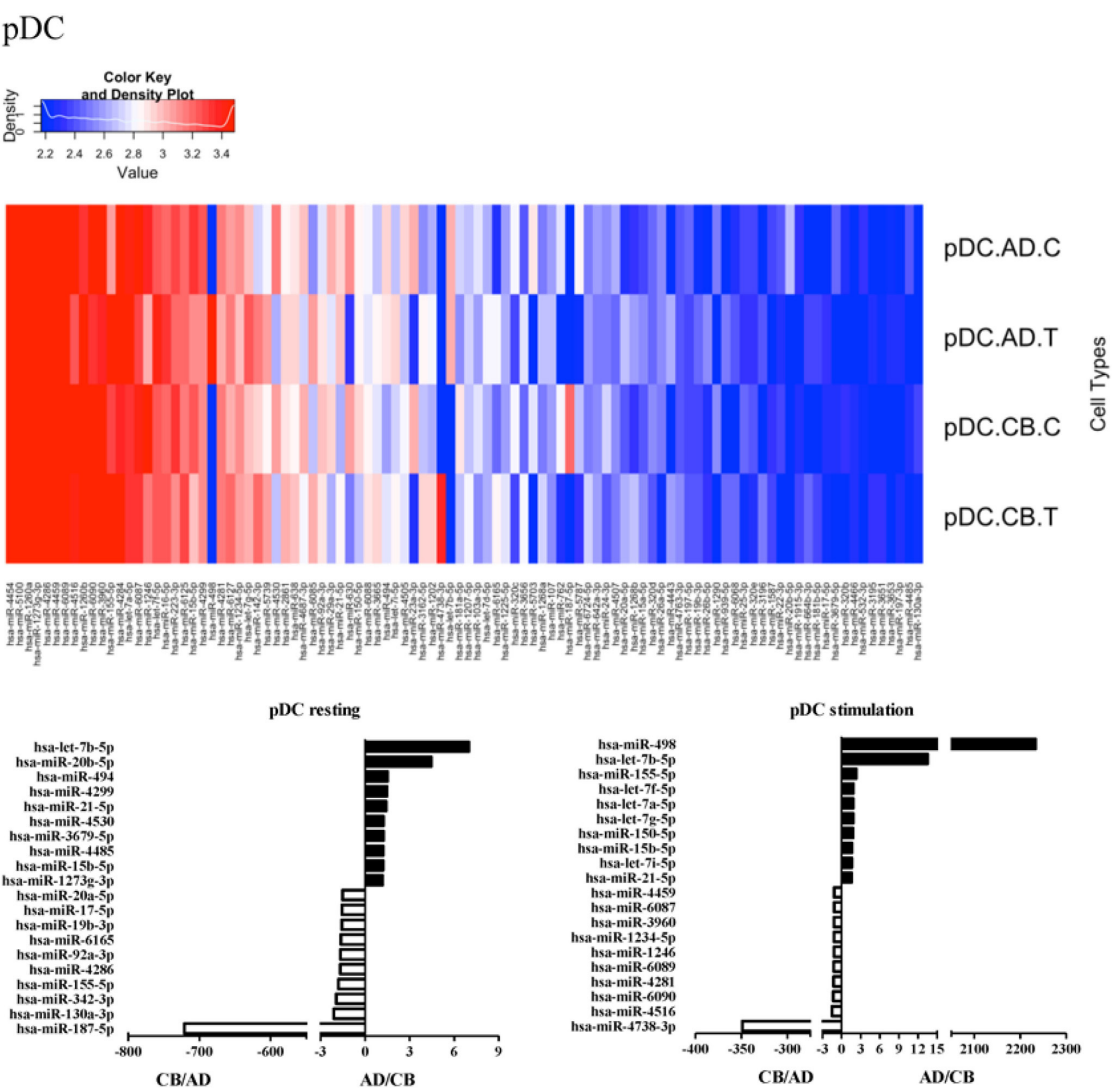
**Differential microRNA profiles between adult and neonatal plasmacytoid dendritic cells**.

Since the major roles of immune cells are to defend the body against foreign substances, stimulation is needed to activate immune responses. Therefore, we also compared the miRNA profiles of leukocyte subsets between adult and neonate samples upon stimulation. Among the differentially expressed miRNA, the top 20 miRNAs expressed in specific leukocytes from adult (more than or less than neonate) samples were chosen and illustrated. The differentially expressed miRNA profiles of PMNs, CD4^+^ T cells, CD8^+^ T cells, CD14^+^ monocytes, CD19^+^ B-cells, CD56^+^ NK cells, mDCs, and pDCs are shown in Figures 3–10, respectively. Under resting conditions, compared to adult counterparts, the top 10 underexpressed miRNAs in CB PMNs were let-7b-5p, let-7c, miR-4516, miR-4466, miR-4281, miR-2861, miR-1225-5p, miR-6090, miR-1234-5p, and miR-3663-3p. The top 10 miRNAs (miR-586, miR-451a, miR-17-5p, miR-145-5p, miR-6127, miR-199a-3p, miR-3135b, miR-423-5p, miR-3651, and miR-93-5p) were overexpressed in CB PMNs (Figure 3). Upon LPS stimulation, compared to adult counterparts, the top 10 underexpressed miRNAs in CB PMNs were miR-142-5p, let-7c, let-7b-5p, miR-101-3p, miR-3676-5p, miR-494, miR-29c-3p, miR-2861, miR-1915-3p, and miR-4466, while the top 10 miRNAs overexpressed in CB PMNs were miR-451a, miR-17-5p, miR-199b-5p, miR-320d, miR-199a-3p, miR-361-5p, miR-342-3b, miR-15b-5p, miR-181a-5p, and miR-20a-5p (Figure 3). With LPS stimulation, miR-142-5p exhibited the highest expression in adult PMNs compared to CB PMNs and miR-451a showed the highest expression in neonatal PMNs compared to adult PMNs. let-7b-5p and miR-142-5p exhibited the highest expression in adult PMNs compared to neonatal PMNs at resting and activated states, respectively. miR-586 and miR-451a exhibited the highest expression in cord PMNs compared to adult PMNs at resting and activated states, respectively. These dynamic changes in miRNA profiles correspond to the functional roles of miRNAs in immune cells during stimulation. Similarly, miR-1238-3p exhibited the highest expression in adult CD4^+^ T cells compared to neonatal CD4^+^ T cells at resting state (Figure 4). miR-19a-5p had the highest expression in neonatal CD4^+^ T cells compared to adult CD4^+^ T-cells at resting state. With PHA stimulation, miR-551b-3p exhibited the highest increase in expression in adult CD4^+^ T cells. miR-518c-3p showed the highest expression in neonatal CD4^+^ T cells compared to adult CD4^+^ T cells after PHA stimulation. Interestingly, let-7b-5p exhibited higher expression in adult monocytes compared to neonatal monocytes with or without stimulation (Figure 6). Likewise, we identified unique miRNA profiles of other leukocyte subsets between adults and neonates with or without stimulation. These unique miRNA profiles between adult and neonate leukocytes likely contribute to the altered immune function of neonates. It is noteworthy that adult peripheral leukocytes consistently demonstrated higher let-7b-5p and lower miR-181a-5p expression than CB cells across multiple subsets, with or without stimulation.

**Figure 11:**
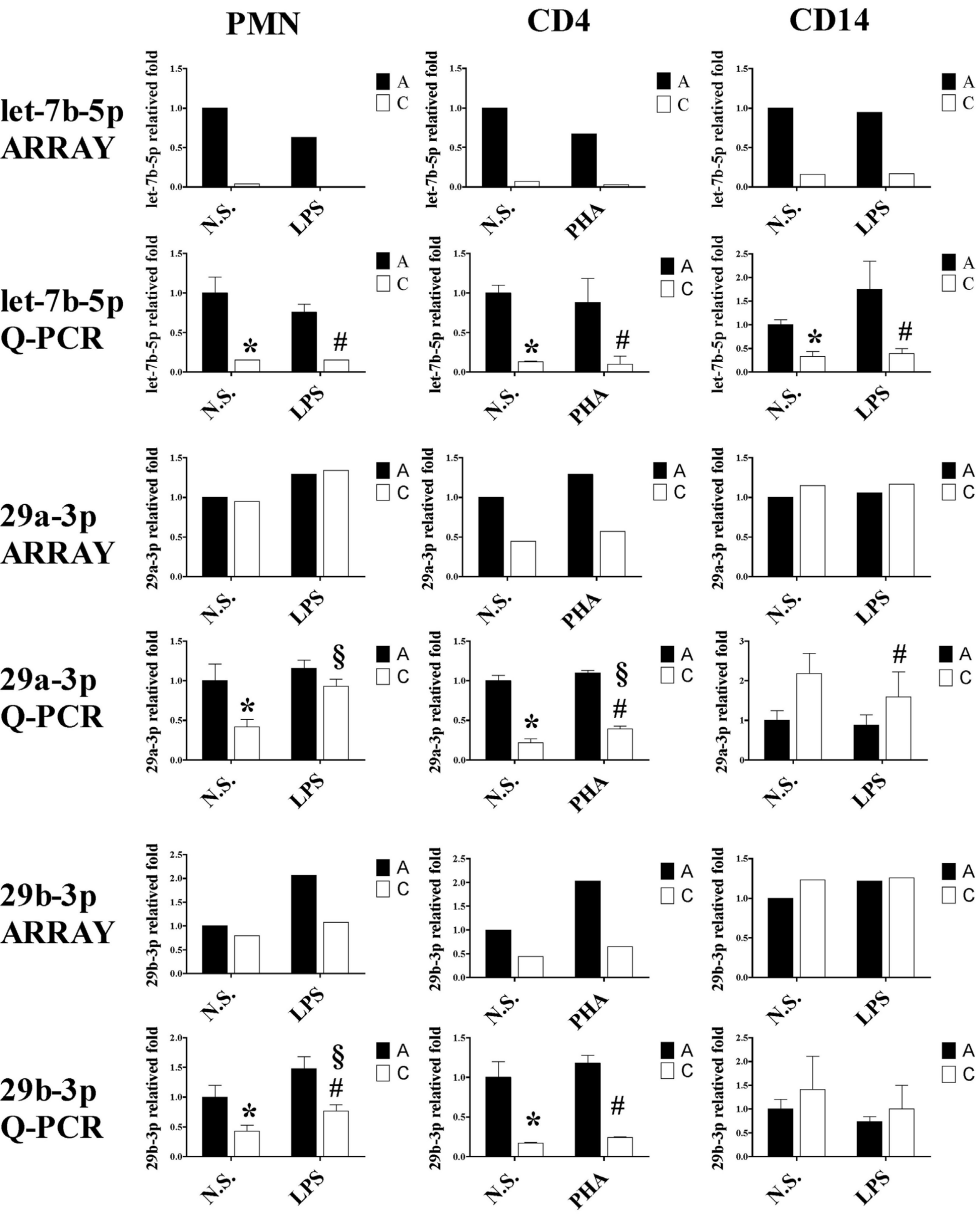

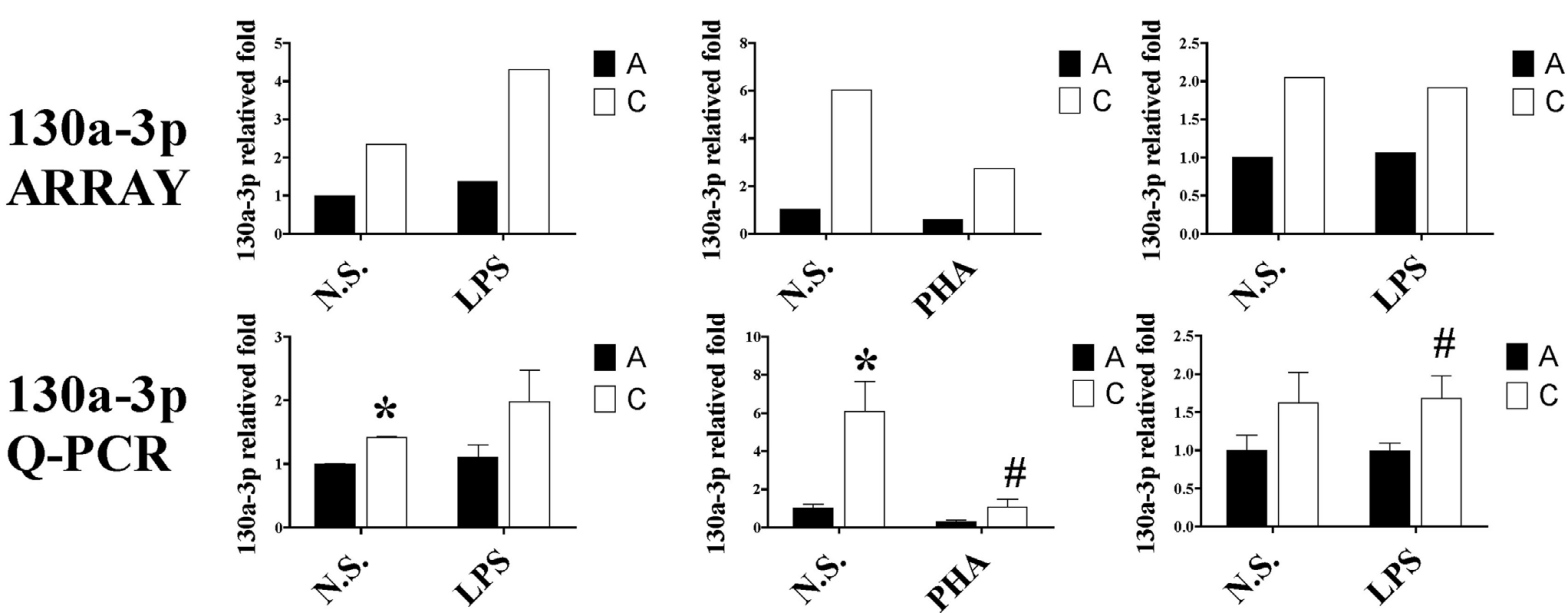
**Quantitative reverse transcription PCR (qRT-PCR) validation of let-7b-5p, 29a-3p, 29b-3p, and miR-130a-3p expression in various leukocyte subsets from cord blood (CB) and adult peripheral blood**. To validate the data derived from our microarray analysis and literature references, we subjected RNA extracts harvested from stimulated or unstimulated polymorphonuclear cell, CD4^+^ T cells, and CD14^+^ monocytes of neonatal and adult samples for qRT-PCR analysis of let-7b-5p, miR-29a-3p, miR-29b-3p, and miR-130a-3p expression *p < 0.05 between indicated adult and CB leukocytes without stimulation; ^#^*p* < 0.05 between indicated adult and CB leukocytes with stimulation; ^§^*p* < 0.05 between indicated CB leukocytes without and with stimulation (by ANOVA with the LSD *post hoc* test, *n* = 4).

Since the differentially expressed miRNAs were selected based on signal intensity from a chip array with pooled leukocytes, we selected let-7b-5p, miR-29a-3p, miR-29b-3p, and miR-130a-3p to validate the array results by qRT-PCR in PMN, CD4^+^ T cells, and CD14^+^ monocytes. As shown in Figure 11, the qRT-PCR results demonstrated a decrease in the expression of miR-29a-3p in PMNs and CD4^+^ T cells of CB compared to adult cells, as opposed to CD14^+^ monocytes. A similar trend to miR-29a-3p was observed with the expression of miR-29b-3p, whereas the expression of miR-130a-3p was higher in neonatal PMN, CD4^+^ T cells, and CD14^+^ cells. These data were consistent with the microarray results.

### Functional Validation of let-7b-5p on Pro-inflammatory Cytokine Production by Monocytes

In the next study, monocytes were selected for further functional validation of let-7b-5p. Because adult monocytes had higher let-7b-5p expression than CB, we transfected a let-7b-5p inhibitor and let-7b-5p mimic into adult monocytes and CB monocytes, respectively. The transfection efficiency of the let-7b-5p inhibitor and mimic were determined by qPCR. As shown in Figure S1 in Supplementary Material, transfection of the let-7b-5p inhibitor into adult monocytes for 24 h suppressed let-7b expression 0.05-fold (at 20 and 40 nM of let-7b-5p inhibitor), white transfection of the let-7b-5p mimic into CB monocytes enhanced the expression of let-7b-5p 16,000- (at 20 nM of let-7b-5p mimic) and 20,000-fold (at 40 nM let-7b-5p mimic).

**Figure 12:**
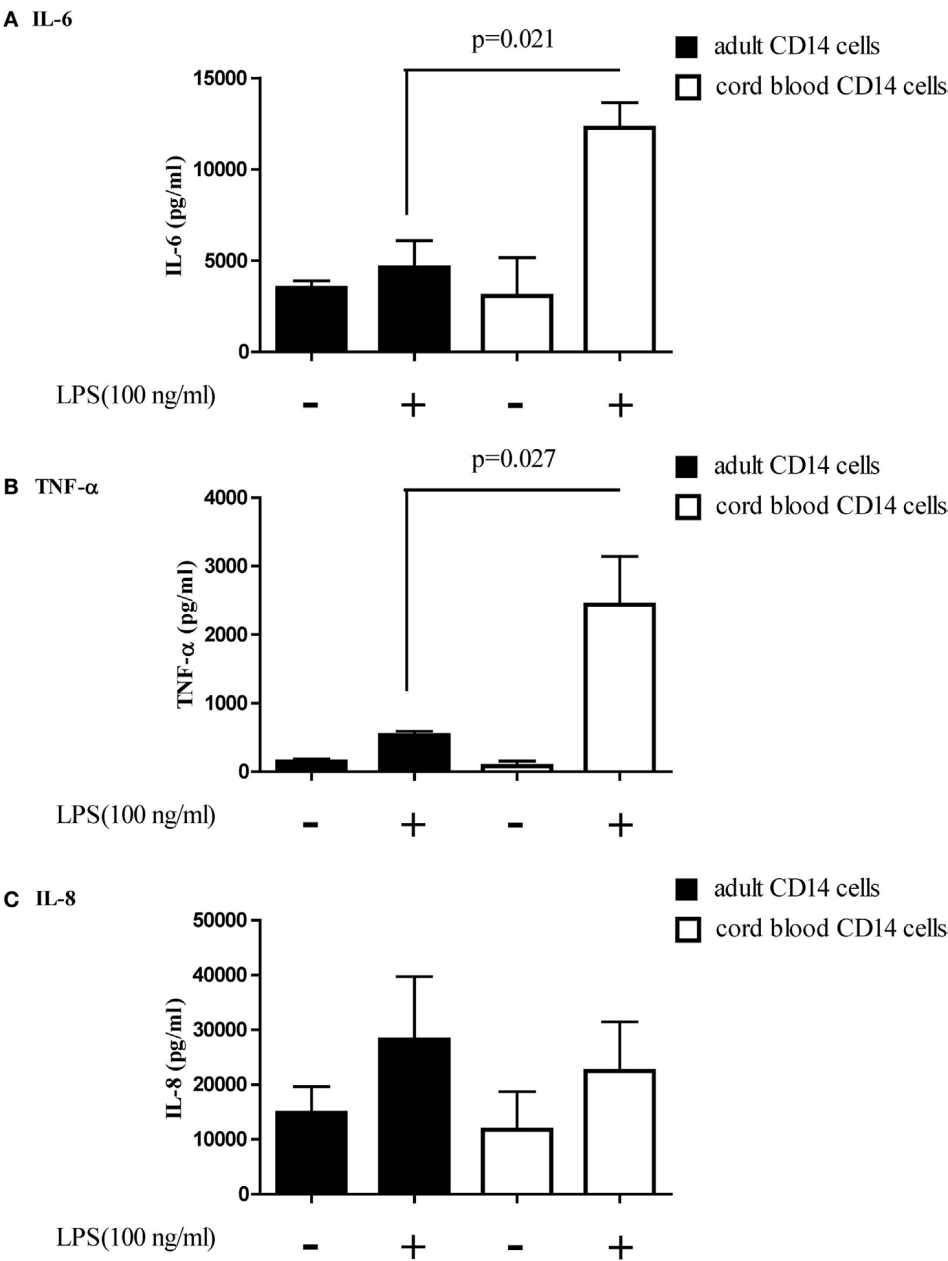
**Adult and cord blood (CB) monocytes produced large amounts of pro-inflammatory cytokines upon LPS stimulation**. Adult and CB monocytes were stimulated with LPS for 16 h, and then the supernatant was collected for quantification of **(A)** interleukin (IL)-6, **(B)** IL-8, and **(C)** TNF-α secretion. *p < 0.05 between adult and CB monocytes with Mann–Whitney *U* test (*n* = 5).

**Figure 13:**
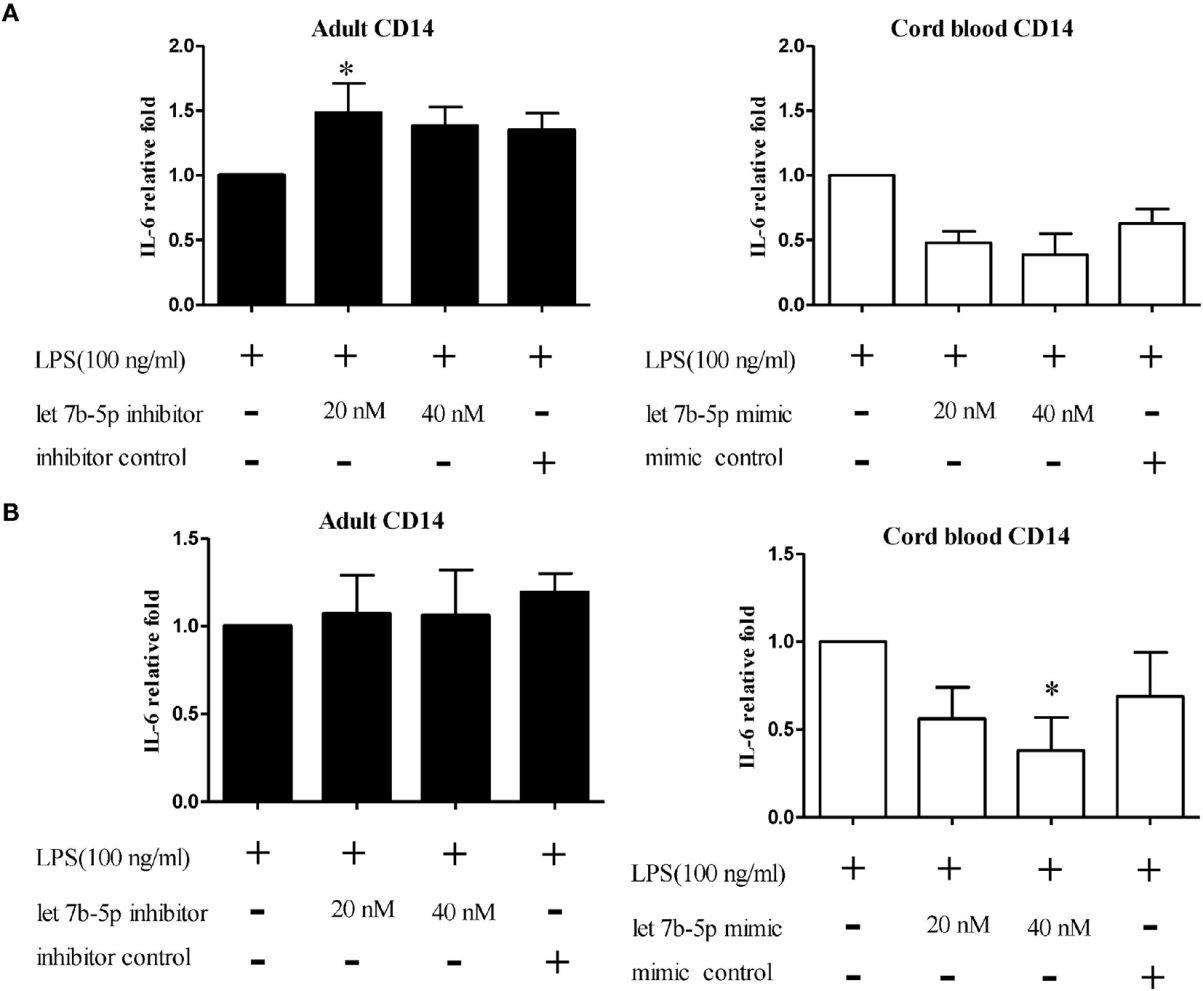
**The effect of let-7b-5p on interleukin (IL)-6 production by monocytes**. let-7b-5p inhibitor transfected or untransfected adult monocytes and let-7b-5p mimic transfected and untransfected CB monocytes were subjected to detection of the IL-6 protein after LPS stimulation for 16 h. The transfection times were **(A)** 24 h and **(B)** 48 h. IL-6 production is represented as relative fold expression compared to the LPS stimulation control. *p < 0.05 as compared with LPS stimulation only with Mann–Whitney *U* test (*n* = 5).

**Figure 14:**
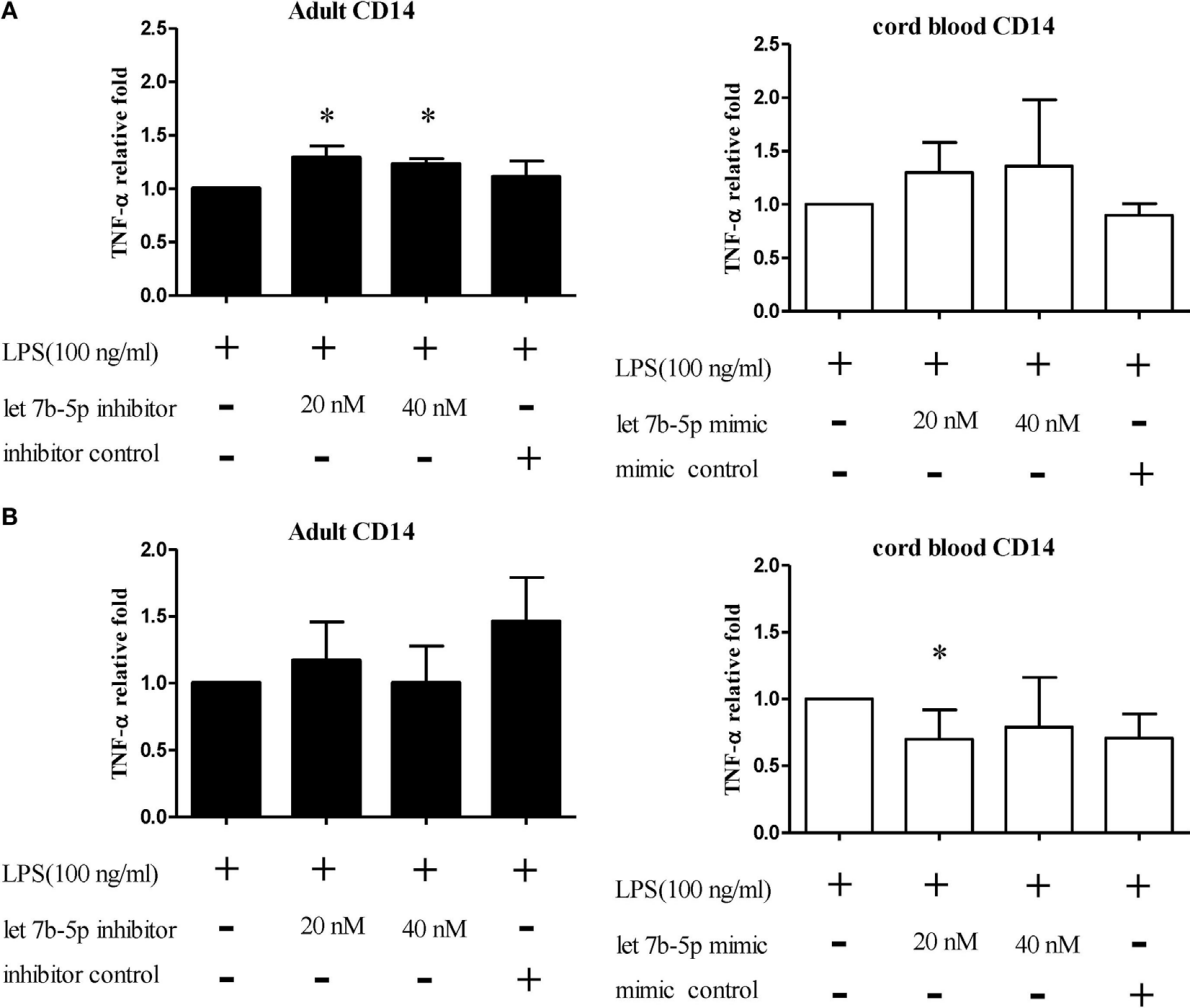
**The effect of let-7b-5p on TNF-α production by monocytes**. Adult monocytes with or without let-7b-5p inhibitor transfection and cord blood monocytes with or without let-7b-5p mimic transfection were subjected to detection of TNF-α protein after LPS stimulation for 16 h. The transfection times were **(A)** 24 h and **(B)** 48 h. TNF-α production is represented as relative fold expression compared to the LPS stimulation control. Data presented are calculated from five replicate measurements. *p < 0.05 as compared with LPS stimulation only with Mann–Whitney *U* test (*n* = 5).

**Figure 15:**
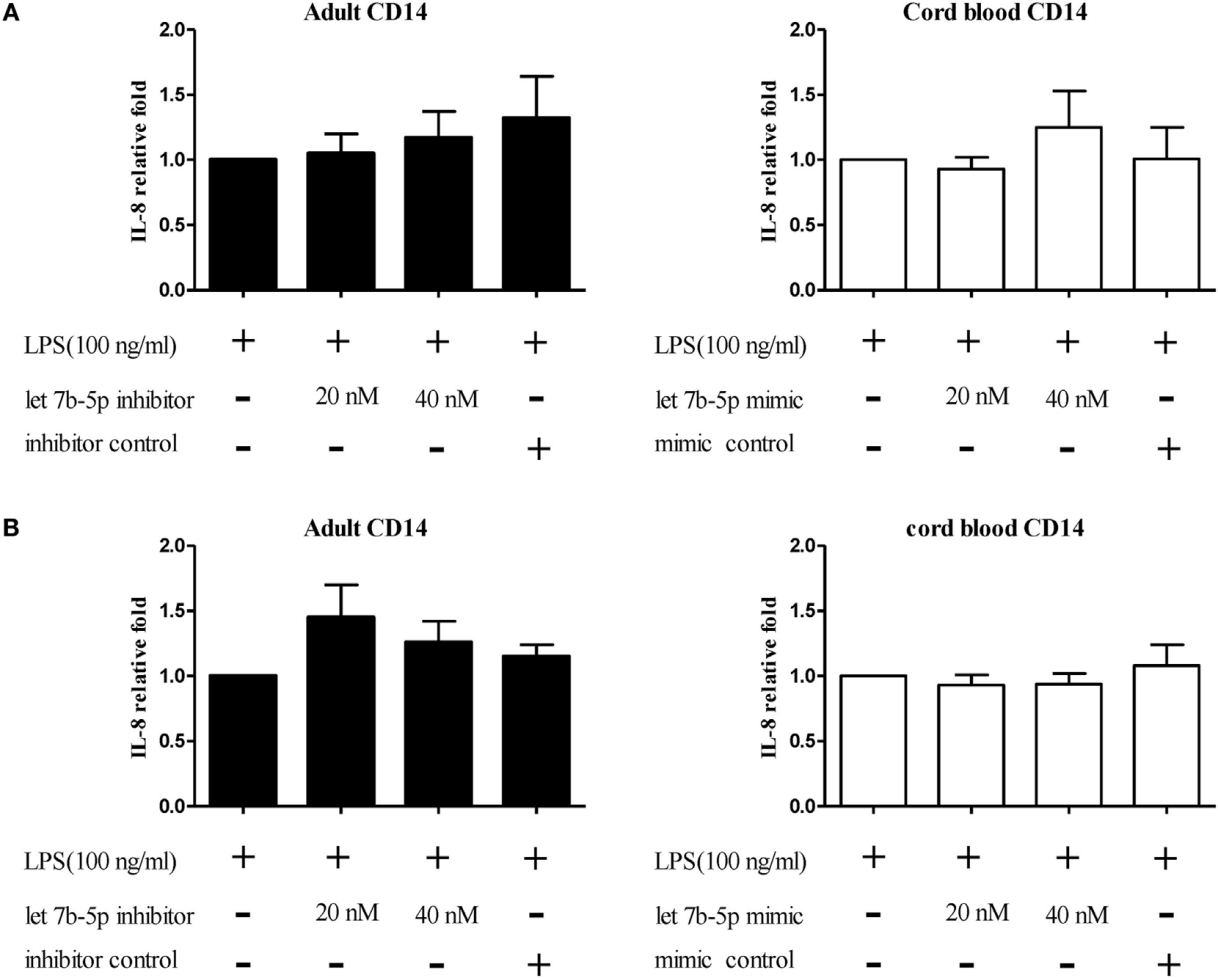
**The effect of let-7b-5p on interleukin (IL)-8 production by monocytes**. Adult monocytes with or without let-7b-5p inhibitor transfection and cord blood monocytes with or without let-7b-5p mimic transfection were subjected to detection of the IL-8 protein after LPS stimulation for 16 h. The transfection times were **(A)** 24 h and **(B)** 48 h. IL-8 production is represented as relative fold expression compared to the LPS stimulation control. *p < 0.05 as compared with LPS stimulation only with Mann–Whitney *U* test (*n* = 5).

The function of let-7b-5p on the pro-inflammatory cytokine production was determined. Upon LPS stimulation, the monocytes produced large amounts of IL-6, IL-8, and TNF-α. Cord blood monocytes showed higher IL-6 (12,265 ± 1,387 vs. 4,623 ± 1,474 pg/ml) and TNF-α (2,436 ± 706 vs. 536 ± 54 pg/ml) production than adult monocytes (Figure 12). Transfection of the let-7b-5p inhibitor (20 nM) into adult monocytes for 24 h enhanced IL-6 production (Figure 13A). Transfecting the let-7b-5p mimic into CB monocytes for 24 h did not significantly suppress IL-6 production, although there was a trend. After transfecting let-7b-5p mimic into CB monocytes for 48 h, IL-6 production was suppressed with 40 nM of let-7b-5p mimic (Figure 13B). Transfection of the let-7b-5p inhibitor at 20 and 40 nM for 24 h enhanced TNF-α production by adult monocytes (Figure 14A). However, transfection of let-7b-5p mimic at 20 nM for 48 h was needed to suppress TNF-α production by CB monocytes (Figure 14B). These results suggest that the modulatory effects of CB monocytes could be achieved with let-7b mimic, but a longer incubation time is needed. Either transfection with the let-7b-5p inhibitor or let-7b-5p mimic for 24 or 48 h had no influence on IL-8 production by adult or CB monocytes (Figures 15A,B).

## Discussion

Blood contains different leukocyte subsets owning distinct gene expression profiles corresponding to distinct immune functions upon stimulation. Since miRNA behavior at the molecular level is context and cell type dependent, knowing the distinct functions of specific mRNAs in adult and neonatal leukocyte subsets can help to clarify the mechanisms leading to altered immunity in newborns. In this study, we conducted a systematic and comprehensive analysis of functional miRNAs in purified PMN, monocytes, T cells, NK cells, B cells, pDCs, and mDCs from neonates and adults. Transfection of the let-7b inhibitor into adult monocytes significantly enhanced IL-6 and TNF-α production, while transfection of a let-7b mimic into CB monocytes significantly suppressed the production of these cytokines. Accordingly, functional validation identified higher IL-6 and TNF-α production by CB monocytes than adult monocytes upon LPS stimulation. This is, at least partly, due to their lower let-7b expression. Our results demonstrate the essential roles of specific miRNAs in regulating neonatal immune functions and provide insight into the molecular mechanism.

Understanding of the immune response in disease is an important issue for living organisms. The immune system is a complex system of interacting cells whose primary purpose is to identify foreign substances. To exhibit proper immune function, the immune cells must undergo activation, proliferation, and cytokine production upon encountering antigens (27). Therefore, activated leukocyte subsets have more biological transcripts than resting cells (3, 23). In order to investigate the corresponding innate or adaptive immune response, leukocyte subsets were isolated and stimulated with LPS, PHA, or CpG ODN as indicated in this study. LPS is a component of Gram-negative bacteria and is used in *in vitro* studies to assess innate immune responses to Gram-negative bacterial infection (32, 33). PHA is a lectin that binds to the sugars on glycosylated surface proteins, including the T cell receptor, and thereby crosslinks T cell activation signals. PHA has been widely used to stimulate human lymphocytes (34), whereas unmethylated CpG ODN is a ligand for TLR9 of pDCs (29). We chose LPS as a stimulus for PMN, monocytes, and mDCs. PHA was used to stimulate lymphocytes and NK cells, while CpG ODN was used to stimulate pDCs. With this approach, we provide functionally differential miRNA expression profiling of specific immune cell subsets between neonates and adults.

Although some studies have reported differences in miRNA expression between cord and adult blood immune cells, most focused on the miRNA profiles of a single resting leukocyte subpopulation (20, 22, 35). Several studies have provided miRNA profiles for limited leukocyte subsets without comparing to adults, thus providing only limited array information (28, 36, 37). Our study identified many previously reported miRNAs as well as unidentified miRNAs in all leukocyte subsets. We determined that all adult leukocyte subsets show higher let-7b-5p expression than the corresponding CB leukocytes, irrespective of their activation state. The let-7 miRNA family has been reported to consistently demonstrate increased abundance in adult erythroid cells (38).

To the best of our knowledge, this study is the first to demonstrate the abundance of let-7b-5p in all adult leukocyte subsets compared to neonates. Our data also demonstrate that neonatal CD4^+^ T cells have more abundant miR-181a expression than adult CD4^+^ T cells as previously reported by Palin et al. (35). Furthermore, our data show that all neonatal leukocyte subsets have more abundant miR-181a expression than relevant cells in adults, besides CD4^+^ T cells. Our findings also correlate with that of Charrier et al. showing that neonatal pDCs have more abundant TLR signal-related miR-155-5p (called miR-155 previously) expression than adult pDCs at the resting state (22). Moreover, we found that miR-155-5p expression in adult pDCs is upregulated and compatible with neonatal pDCs after CPG ODN stimulation. Our study confirms the overexpression of miR-15b, miR-181a, miR-363, and miR-424 in CB CD4^+^ and CD8^+^ T cells, as previously reported (37). However, we did not observe significant change in miR-155 expression after LPS stimulation in our array data, as reported by Takahashi et al. (37). The dynamic changes of miR-155 presented in our previous study could explain this inconsistency (23). Higher miR-184 expression in CB CD4^+^ T cells that regulate the abundance of NFAT1 has been reported (20). However, our data showed low miR-184 expression below the threshold setting, in adult and cord CD4^+^ T cells. Three small-RNA transcripts were expressed specifically in one cell type: miR-378 in monocytes, miR-31 in T cells, and miR-143 in neutrophils (25). In our study, we did observe specific miR-143 in neutrophils, although miR-378 and miR-31 were selectively expressed in adult and neonatal monocytes and T cells, respectively. These inconsistent results in miRNA expression among the different studies might be due to the differences of array or isolation kits used for study.

Based on our data, all adult leukocyte subsets illustrated higher let-7b-5p expression than the corresponding CB leukocytes. The higher let-7b-5p expression in adult leukocyte subsets was 3.7- to 63.0-fold that of neonates (Table S1 in Supplementary Material). PHA-stimulated adult CD4^+^ T cells and CD8^+^ T cells had higher let-7b-5p expression than neonates (63.0- and 40.2-fold, respectively).

let-7 is an important miRNA family consisting of 13 members. They are highly conserved across several animal species (39). The role of let-7 family members in tumor suppression is well established, and their role in the regulation of innate immunity is slowly being unveiled. Teng et al. have shown that let-7b regulates the expression of TLR4 *via* posttranscriptional suppression and subsequently influences the activation of NF-κB and downstream gene expression (40). Type 1 IFN production and antiviral signaling cascades are an important defense mechanism in the innate immune response to viral infections. let-7b has also been shown to regulate type 1 IFN production and can inhibit HCV replication and viral protein translation through insulin-like growth factor 2 mRNA-binding protein 1 (41–43). Whether diminished let-7b expression in neonatal leukocyte subsets can lead to altered type 1 IFN production and the susceptibility of newborns to viral infections need further investigation (1, 29). In our study, let-7b-5p modulated IL-6 and TNF-α production by monocytes. It has been suggested that, with LPS stimulation, hepatocarcinoma cells transfect let-7b enriched microparticles into macrophages, resulting in IL-6 downregulation in macrophages (44). We observed that more abundant let-7b expression in adult monocytes corresponded to lower IL-6 production than in CB monocytes with LPS stimulation. Transfection of adult monocytes with a let-7b-5p inhibitor enhanced IL-6 production, while transfection of a let-7b-5p mimic into CB monocytes suppressed IL-6 production. This suggests that reduced let-7b expression in CB monocytes contributes to higher IL-6 production, compared to adult monocytes. Since IL-6 can modulate the recruitment and differentiation of T and B lymphocytes (45), let-7b plays an important modulatory role in the altered immune responses in newborns.

Interleukin-6 is a multifunctional cytokine secreted by many kinds of cells. IL-6 is produced in response to TLR ligands and other pro-inflammatory cytokines through the NF-κB signaling pathway (45). Although let-7b was also reported to target TLR4 and suppress IL-8 production through NF-κB regulation in gastric epithelial cells with *Helicobacter pylori* infection (40), there are likely different mechanisms for IL-6/IL-8 production control in specific cells, as let-7b cannot influence IL-8 production in both adult and CB monocytes.

Neonates are susceptible to infection, and neonatal sepsis is a significant cause of mortality and morbidity in newborns (46). Inflammatory responses are necessary for the host to respond to components of invading organisms. At the early stages of infection, TNF-α, IL-1β, IL-6, and IL-8 pro-inflammatory cytokines are produced and stimulate immune cells leading to activation of an inflammatory cascade (47). The subsequent inflammatory cascade includes many biologically active mediators and can induce a systemic inflammatory response. The inflammatory response is thereafter counteracted by anti-inflammatory mediators to restore immunological homeostasis. Excessive pro-inflammatory mediators or inadequate anti-inflammatory responses will lead to sepsis (47). In addition to antibiotics, other adjuvant therapies primarily target the dysregulated innate immune response. Pentoxifylline, a xanthine-derived phosphodiesterase inhibitor that decreases TNF-α and IL-6 production, has been reported to improve the clinical outcome of preterm sepsis (48). Recently, anti-cytokine agents in early sepsis with excessive uncontrolled hyper-inflammatory state have been addressed (49). Riedemann et al. demonstrated that an anti-IL-6 antibody could significantly improve survival through reduction of C5aR in various organs in a cecal ligation/puncture model in mice (50). Remick et al. also showed that the combination of IL-1 receptor antagonist and TNF soluble receptor is also effective in this clinically relevant mouse model of sepsis (51). Although each strategy has certain benefits, there is still controversy regarding the general clinical application and potential side effects. Since transfection of let-7b-5p mimic could suppress IL-6 and TNF-α production of CB monocytes, regulation of let-7b-5p has the potential to play a role in neonatal sepsis control.

In conclusion, we demonstrate systematic and functional miRNA profiles of neonate and adult leukocyte subsets. We show that all adult leukocyte subsets have higher let-7b-5p expression than the corresponding CB leukocytes. let-7b-5p negatively regulates the production of IL-6 and TNF-α by human monocytes. Our study supports a comprehensive miRNA transcriptome database of neonatal immune cells and serves as a valuable resource for elucidating the role of miRNA-mediated regulation in neonatal immunity.

## Author Contributions

H-RY, T-YH, H-CH, H-CK, and S-CL contributed to designed the work; H-RY, T-YH, H-CH, H-CK, S-CL, and K-SH contributed to data acquisition; H-RY, T-YH, H-CH, H-CK, and S-CL performed data analysis and interpretation; H-RY, KY, and K-SH drafted the manuscript; H-RY and T-YH finalized the article. All the authors have read and approved the final manuscript and agreed to be accountable for all aspects of the work.

## Conflict of Interest Statement

The authors declare that the research was conducted in the absence of any commercial or financial relationships that could be construed as a potential conflict of interest.

## Supplementary Material

Figure S1 **Transfection of let-7b-5p inhibitor and mimic into adult and cord blood (CB) monocytes, respectively**. **(A)** Transfection of the let-7b-5p inhibitor into adult monocytes suppressed the expression of let-7b-5p. **(B)** Transfection of let-7b-5p mimic into CB monocytes enhanced the expression of let-7b-5p.
