# Supplementary material for: Comparison of the Functional microRNA Expression in Immune Cell Subsets of Neonates and Adults
Source: Front Immunol. 2016 Dec 19;7:615. doi: 10.3389/fimmu.2016.00615 (PMC5165026; doi:10.3389/fimmu.2016.00615)
Supplement: Supplementary file 2 [file Table_2.DOC]

Supplementary Table 2. Relative miRNA expression among the different leukocytes subsets of adult and cord blood samples
