# Supplementary material for: Comparison of the Functional microRNA Expression in Immune Cell Subsets of Neonates and Adults
Source: Front Immunol. 2016 Dec 19;7:615. doi: 10.3389/fimmu.2016.00615 (PMC5165026; doi:10.3389/fimmu.2016.00615)
Supplement: Supplementary file 4 [file Table_4.DOC]

Supplementary Table 4. Leukocytes miRNA signature before and after stimulation

| PMN-C | | PMN-T | |
| --- | --- | --- | --- |
| micro RNA | CB/AD | micro RNA | CB/AD |
| | hsa-miR-586 | | --- | | hsa-miR-451a | | hsa-miR-17-5p | | hsa-miR-145-5p | | hsa-miR-6127 | | hsa-miR-199a-3p | | hsa-miR-3135b | | hsa-miR-423-5p | | hsa-miR-3651 | | hsa-miR-93-5p | | | 267.2 | | --- | | 4.4 | | 1.9 | | 1.8 | | 1.8 | | 1.7 | | 1.7 | | 1.7 | | 1.7 | | 1.6 | | | hsa-miR-15b-5p | | --- | | hsa-miR-17-5p | | hsa-miR-181a-5p | | hsa-miR-199a-3p | | hsa-miR-199b-5p | | hsa-miR-20a-5p | | hsa-miR-320d | | hsa-miR-342-3p | | hsa-miR-361-5p | | hsa-miR-451a | | | 7.74 | | --- | | 2.22 | | 2.21 | | 2.16 | | 2.06 | | 2.02 | | 1.99 | | 1.89 | | 1.89 | | 1.81 | |
| micro RNA | AD/CB | micro RNA | AD/CB |
| | hsa-miR-3663-3p | | --- | | hsa-miR-1234-5p | | hsa-miR-6090 | | hsa-miR-1225-5p | | hsa-miR-2861 | | hsa-miR-4281 | | hsa-miR-4466 | | hsa-miR-4516 | | hsa-let-7c | | hsa-let-7b-5p | | | 1.5 | | --- | | 1.6 | | 1.6 | | 1.6 | | 1.6 | | 1.7 | | 1.7 | | 1.7 | | 3.9 | | 4.9 | | | hsa-miR-4466 | | --- | | hsa-miR-1915-3p | | hsa-miR-2861 | | hsa-miR-29c-3p | | hsa-miR-494 | | hsa-miR-3676-5p | | hsa-miR-101-3p | | hsa-let-7b-5p | | hsa-let-7c | | hsa-miR-142-5p | | | 1.96 | | --- | | 1.96 | | 1.97 | | 2.08 | | 2.18 | | 2.74 | | 2.83 | | 3.16 | | 3.17 | | 3.74 | |

PMN

CD4

| CD4-C | | CD4-T | |
| --- | --- | --- | --- |
| micro RNA | CB/AD | micro RNA | CB/AD |
| | hsa-miR-19a-5p | | --- | | hsa-miR-3156-5p | | hsa-miR-130a-3p | | hsa-miR-363-3p | | hsa-miR-1305 | | hsa-miR-424-5p | | hsa-miR-6131 | | hsa-miR-181a-5p | | hsa-miR-148a-3p | | hsa-miR-151a-5p | | | 578.8 | | --- | | 7.6 | | 6 | | 5.4 | | 4.3 | | 3.8 | | 3.1 | | 2.8 | | 2.6 | | 2.4 | | | hsa-miR-518c-3p | | --- | | hsa-miR-1206 | | hsa-miR-363-3p | | hsa-miR-424-5p | | hsa-miR-181a-5p | | hsa-miR-4713-3p | | hsa-miR-15b-5p | | hsa-miR-17-5p | | hsa-miR-93-5p | | hsa-miR-18a-5p | | | 979.97 | | --- | | 177.93 | | 2.68 | | 2.17 | | 2.08 | | 2.01 | | 1.6 | | 1.47 | | 1.45 | | 1.38 | |
| micro RNA | AD/CB | micro RNA | AD/CB |
| | hsa-miR-5703 | | --- | | hsa-miR-630 | | hsa-miR-4459 | | hsa-miR-642a-3p | | hsa-let-7c | | hsa-miR-762 | | hsa-miR-5787 | | hsa-miR-186-5p | | hsa-let-7b-5p | | hsa-miR-1238-3p | | | 3.42 | | --- | | 3.79 | | 4.02 | | 4.11 | | 4.31 | | 4.83 | | 5.34 | | 11.68 | | 21.52 | | 45.42 | | | hsa-let-7c | | --- | | hsa-miR-4763-3p | | hsa-miR-630 | | hsa-miR-4741 | | hsa-miR-3663-3p | | hsa-miR-642a-3p | | hsa-miR-762 | | hsa-miR-5787 | | hsa-let-7b-5p | | hsa-miR-551b-3p | | | 6.93 | | --- | | 7.03 | | 8.15 | | 8.89 | | 9.46 | | 9.47 | | 11.11 | | 11.41 | | 44.28 | | 1376.66 | |

CD8

| CD8-C | | CD8-T | |
| --- | --- | --- | --- |
| micro RNA | CB/AD | micro RNA | CB/AD |
| | hsa-miR-424-5p | | --- | | hsa-miR-3135b | | hsa-miR-223-3p | | hsa-miR-15b-5p | | hsa-miR-363-3p | | hsa-miR-15a-5p | | hsa-miR-181b-5p | | hsa-miR-146a-5p | | hsa-miR-16-5p | | hsa-miR-574-5p | | | 7.9 | | --- | | 7.2 | | 4.8 | | 4.2 | | 4.1 | | 3.8 | | 3.8 | | 3.8 | | 3.7 | | 3.2 | | | hsa-miR-424-5p | | --- | | hsa-miR-146a-5p | | hsa-miR-223-3p | | hsa-miR-363-3p | | hsa-miR-15a-5p | | hsa-miR-15b-5p | | hsa-miR-155-5p | | hsa-miR-16-5p | | hsa-miR-181a-5p | | hsa-miR-181b-5p | | | 6.75 | | --- | | 6.16 | | 3.96 | | 3.86 | | 3.46 | | 3.43 | | 3.37 | | 3.23 | | 3.19 | | 3.13 | |
| micro RNA | AD/CB | micro RNA | AD/CB |
| hsa-miR-6085  hsa-miR-21-5p  hsa-miR-101-3p  hsa-miR-1260a  hsa-miR-29a-3p  hsa-miR-29b-3p  hsa-miR-1260b  hsa-miR-29c-3p  hsa-let-7c  hsa-let-7b-5p | | 1.47 | | --- | | 1.52 | | 1.56 | | 1.65 | | 1.69 | | 1.73 | | 1.85 | | 3.43 | | 4.79 | | 30.74 | | | hsa-miR-664b-3p | | --- | | hsa-miR-4284 | | hsa-miR-29b-3p | | hsa-miR-3653 | | hsa-miR-1260b | | hsa-miR-101-3p | | hsa-miR-29a-3p | | hsa-miR-29c-3p | | hsa-let-7c | | hsa-let-7b-5p | | | 1.52 | | --- | | 1.56 | | 1.57 | | 1.58 | | 1.6 | | 1.73 | | 1.93 | | 4.06 | | 4.49 | | 32.3 | |

CD14

| CD14-C | | CD14-T | |
| --- | --- | --- | --- |
| micro RNA | CB/AD | micro RNA | CB/AD |
| hsa-miR-3156-5p  hsa-miR-1305  hsa-miR-34a-5p  hsa-miR-582-5p  hsa-miR-6131  hsa-miR-199b-5p  hsa-miR-199a-3p  hsa-miR-130a-3p  hsa-miR-1914-3p  hsa-miR-424-5p | | 4.9 | | --- | | 3 | | 2.6 | | 2.6 | | 2.5 | | 2.4 | | 2.1 | | 2 | | 2 | | 1.8 | | | hsa-miR-5590-3p | | --- | | hsa-miR-3156-5p | | hsa-miR-34a-5p | | hsa-miR-582-5p | | hsa-miR-199b-5p | | hsa-miR-199a-3p | | hsa-miR-130a-3p | | hsa-miR-4713-3p | | hsa-miR-424-5p | | hsa-miR-4716-3p | | | 470.55 | | --- | | 2.75 | | 2.36 | | 2.34 | | 2.27 | | 2.1 | | 1.8 | | 1.73 | | 1.68 | | 1.63 | |
| micro RNA | AD/CB | micro RNA | AD/CB |
| hsa-miR-320c  hsa-miR-4741  hsa-miR-5703  hsa-miR-4672  hsa-miR-4459  hsa-miR-1275  hsa-miR-630  hsa-miR-5787  hsa-let-7c  hsa-let-7b-5p | | 2.26 | | --- | | 2.27 | | 2.33 | | 2.4 | | 2.4 | | 2.43 | | 2.65 | | 2.95 | | 3.99 | | 5.11 | | | hsa-miR-762 | | --- | | hsa-miR-1275 | | hsa-miR-4270 | | hsa-miR-6510-5p | | hsa-miR-320c | | hsa-miR-630 | | hsa-miR-5787 | | hsa-miR-6722-3p | | hsa-let-7c | | hsa-let-7b-5p | | | 2.15 | | --- | | 2.21 | | 2.25 | | 2.32 | | 2.35 | | 2.35 | | 2.74 | | 3.26 | | 3.94 | | 4.93 | |

CD19

| CD19-C | | CD19-T | |
| --- | --- | --- | --- |
| micro RNA | CB/AD | micro RNA | CB/AD |
| hsa-miR-98-5p  hsa-miR-4299  hsa-miR-197-5p  hsa-miR-4485  hsa-miR-1587  hsa-miR-4497  hsa-miR-3679-5p  hsa-miR-4505  hsa-miR-181a-5p  hsa-miR-4507 | | 18 | | --- | | 6.7 | | 4.6 | | 4.3 | | 3.8 | | 3.8 | | 3.8 | | 3.8 | | 3.6 | | 3.5 | | | hsa-miR-32-3p | | --- | | hsa-miR-525-5p | | hsa-miR-378h | | hsa-miR-4736 | | hsa-miR-6500-5p | | hsa-miR-4299 | | hsa-miR-197-5p | | hsa-miR-5787 | | hsa-miR-3679-5p | | hsa-miR-642a-3p | | | 728.41 | | --- | | 580.18 | | 438.69 | | 267.96 | | 150.42 | | 4.57 | | 3.53 | | 3.48 | | 3.28 | | 3.25 | |
| micro RNA | AD/CB | micro RNA | AD/CB |
| hsa-miR-19b-3p  hsa-let-7g-5p  hsa-miR-26b-5p  hsa-miR-29a-3p  hsa-miR-29b-3p  hsa-miR-101-3p  hsa-miR-29c-3p  hsa-let-7c  hsa-miR-21-5p  hsa-let-7b-5p | | 1.66 | | --- | | 1.7 | | 1.81 | | 2.26 | | 2.63 | | 2.64 | | 4.07 | | 4.26 | | 4.47 | | 7.6 | | | hsa-miR-6718-5p | | --- | | hsa-miR-513a-5p | | hsa-miR-2117 | | hsa-miR-335-3p | | hsa-miR-4461 | | hsa-miR-3121-5p | | hsa-miR-5580-5p | | hsa-miR-4772-5p | | hsa-miR-4685-5p | | hsa-miR-5591-3p | | | 113.03 | | --- | | 147.61 | | 384.06 | | 415.91 | | 437.73 | | 2296.42 | | 3448.79 | | 3607.04 | | 3839.91 | | 5172.08 | |

CD56

| CD56-C | | CD56-T | |
| --- | --- | --- | --- |
| micro RNA | CB/AD | micro RNA | CB/AD |
| hsa-miR-363-3p  hsa-miR-1268a  hsa-miR-20b-5p  hsa-miR-1246  hsa-miR-1290  hsa-miR-181a-5p  hsa-miR-4299  hsa-miR-197-5p  hsa-miR-17-5p  hsa-miR-181b-5p | | 5.3 | | --- | | 3.2 | | 3 | | 3 | | 3 | | 3 | | 2.8 | | 2.2 | | 2.2 | | 2.1 | | | hsa-miR-3202 | | --- | | hsa-miR-628-3p | | hsa-miR-548az-3p | | hsa-miR-363-3p | | hsa-miR-181a-5p | | hsa-miR-20b-5p | | hsa-miR-181b-5p | | hsa-miR-4299 | | hsa-miR-1246 | | hsa-miR-223-3p | | | 1582.29 | | --- | | 631.62 | | 366.16 | | 6.03 | | 3.47 | | 3.21 | | 2.91 | | 2.61 | | 2.6 | | 2.48 | |
| micro RNA | AD/CB | micro RNA | AD/CB |
| hsa-miR-21-5p  hsa-miR-22-3p  hsa-miR-155-5p  hsa-miR-4284  hsa-miR-29a-3p  hsa-miR-150-5p  hsa-miR-29c-3p  hsa-miR-29b-3p  hsa-let-7c  hsa-let-7b-5p | | 1.38 | | --- | | 1.4 | | 1.45 | | 1.47 | | 1.51 | | 1.63 | | 1.69 | | 1.73 | | 5.59 | | 12.55 | | | hsa-miR-23a-3p | | --- | | hsa-miR-4284 | | hsa-miR-29a-3p | | hsa-miR-155-5p | | hsa-miR-29c-3p | | hsa-miR-21-5p | | hsa-miR-29b-3p | | hsa-let-7b-5p | | hsa-miR-2277-5p | | hsa-miR-6509-5p | | | 1.26 | | --- | | 1.35 | | 1.39 | | 1.41 | | 1.51 | | 1.51 | | 1.78 | | 8.85 | | 3383.37 | | 3641.61 | |

mDC

| mDC-C | | mDC-T | |
| --- | --- | --- | --- |
| micro RNA | CB/AD | micro RNA | CB/AD |
| hsa-miR-451a  hsa-miR-142-5p  hsa-miR-4485  hsa-miR-142-3p  hsa-miR-548n  hsa-miR-4466  hsa-miR-3663-3p  hsa-miR-1915-3p  hsa-miR-19a-3p  hsa-miR-5787 | | 29.02 | | --- | | 11.92 | | 8.17 | | 7.25 | | 6.95 | | 4.18 | | 4.05 | | 4.03 | | 4 | | 3.96 | | | hsa-miR-6507-3p | | --- | | hsa-miR-324-3p | | hsa-miR-22-3p | | hsa-miR-19b-3p | | hsa-miR-642b-3p | | hsa-miR-4741 | | hsa-miR-222-3p | | hsa-miR-664b-3p | | hsa-miR-6131 | | hsa-miR-3651 | | | 147.87 | | --- | | 10.96 | | 8.1 | | 6.37 | | 5.8 | | 5.68 | | 4.86 | | 4.78 | | 4.68 | | 4.5 | |
| micro RNA | AD/CB | micro RNA | AD/CB |
| hsa-miR-4320  hsa-miR-764  hsa-miR-4677-3p  hsa-miR-5591-3p  hsa-miR-5692a  hsa-miR-4726-3p  hsa-miR-3927-3p  hsa-miR-384  hsa-miR-606  hsa-miR-5692c | | 437.06 | | --- | | 439.38 | | 565.74 | | 1874.04 | | 3347.67 | | 4286.54 | | 8750.9 | | 8805.98 | | 9528.61 | | 14398.02 | | | hsa-miR-223-3p | | --- | | hsa-miR-6085 | | hsa-miR-1246 | | hsa-miR-150-5p | | hsa-miR-21-5p | | hsa-miR-16-5p | | hsa-miR-5703 | | hsa-miR-23a-3p | | hsa-miR-29b-3p | | hsa-miR-3960 | | | 1.23 | | --- | | 1.29 | | 1.31 | | 1.46 | | 1.48 | | 1.7 | | 3.96 | | 371.46 | | 537.8 | | 556.44 | |

pDC

| pDC-C | | pDC-T | |
| --- | --- | --- | --- |
| micro RNA | CB/AD | micro RNA | CB/AD |
| hsa-miR-187-5p  hsa-miR-130a-3p  hsa-miR-342-3p  hsa-miR-155-5p  hsa-miR-4286  hsa-miR-92a-3p  hsa-miR-6165  hsa-miR-19b-3p  hsa-miR-17-5p  hsa-miR-20a-5p | | 720.96 | | --- | | 2.11 | | 1.94 | | 1.79 | | 1.67 | | 1.66 | | 1.62 | | 1.57 | | 1.56 | | 1.51 | | | hsa-miR-4738-3p | | --- | | hsa-miR-4516 | | hsa-miR-6090 | | hsa-miR-4281 | | hsa-miR-6089 | | hsa-miR-1246 | | hsa-miR-1234-5p | | hsa-miR-3960 | | hsa-miR-6087 | | hsa-miR-4459 | | | 392.22 | | --- | | 1.69 | | 1.48 | | 1.42 | | 1.37 | | 1.33 | | 1.33 | | 1.32 | | 1.31 | | 1.31 | |
| micro RNA | AD/CB | micro RNA | AD/CB |
| hsa-miR-1273g-3p  hsa-miR-15b-5p  hsa-miR-4485  hsa-miR-3679-5p  hsa-miR-4530  hsa-miR-21-5p  hsa-miR-4299  hsa-miR-494  hsa-miR-20b-5p  hsa-let-7b-5p | | 1.17 | | --- | | 1.21 | | 1.21 | | 1.23 | | 1.23 | | 1.41 | | 1.47 | | 1.52 | | 4.46 | | 8.07 | | | hsa-miR-21-5p | | --- | | hsa-let-7i-5p | | hsa-miR-15b-5p | | hsa-miR-150-5p | | hsa-let-7g-5p | | hsa-let-7a-5p | | hsa-let-7f-5p | | hsa-miR-155-5p | | hsa-let-7b-5p | | hsa-miR-498 | | | 1.39 | | --- | | 1.44 | | 1.45 | | 1.57 | | 1.61 | | 1.63 | | 1.64 | | 2.04 | | 12.01 | | 1987.02 | |

Abbreviations: AD-C: adult blood cells without stimulation; CB-C: cord blood cells without stimulation; AD-T: adult blood cells with stimulation; CB-T: cord blood cells with stimulation.
