# Supplementary figures and images for: Comparison of the Functional microRNA Expression in Immune Cell Subsets of Neonates and Adults
Source: Front Immunol. 2016 Dec 19;7:615. doi: 10.3389/fimmu.2016.00615 (PMC5165026; doi:10.3389/fimmu.2016.00615)

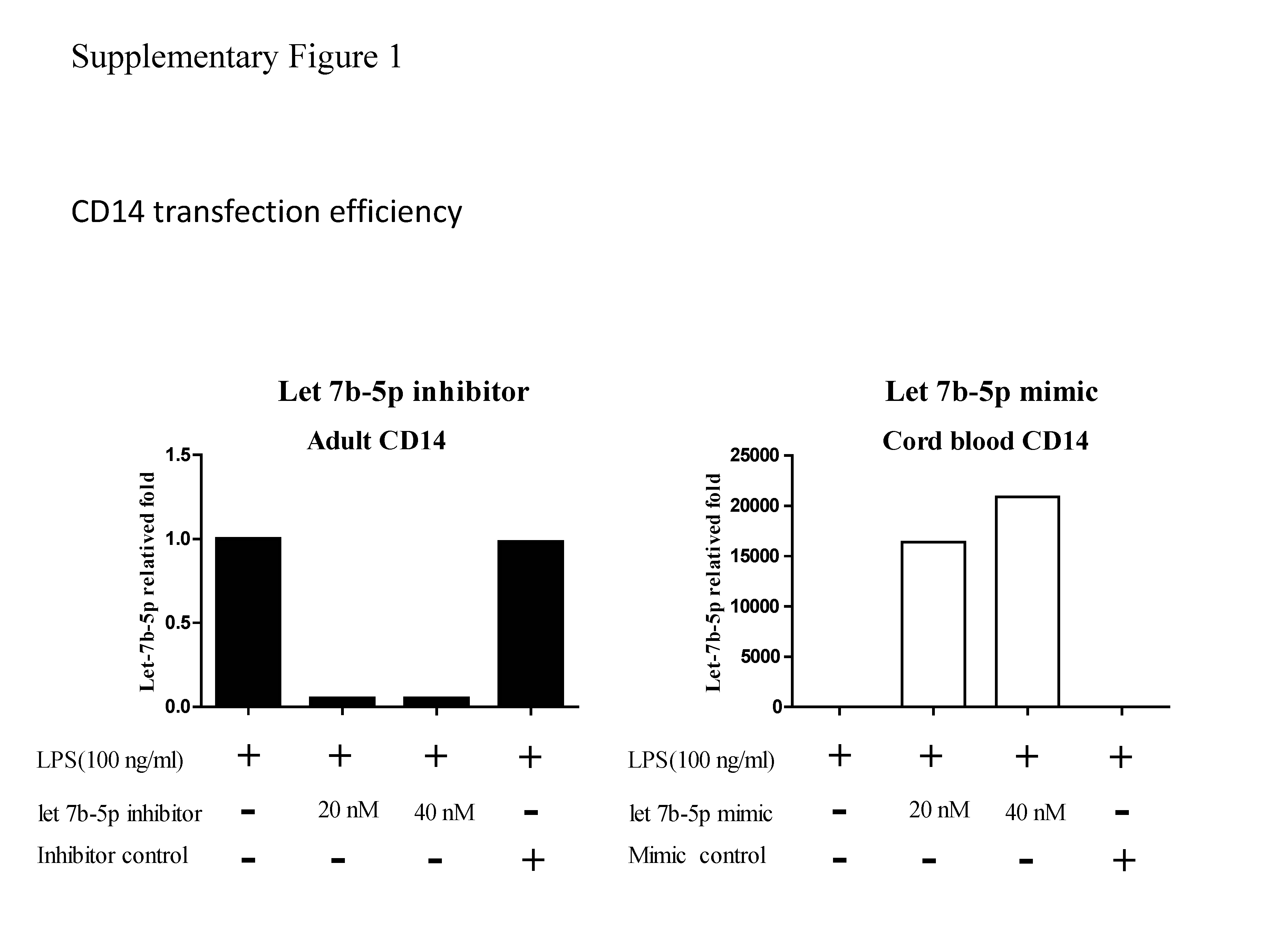

Supplement: Figure S1 — Transfection of let-7b-5p inhibitor and mimic into adult and cord blood (CB) monocytes, respectively. (A) Transfection of the let-7b-5p inhibitor into adult monocytes suppressed the expression of let-7b-5p. (B) Transfection of let-7b-5p mimic into CB monocytes enhanced the expression of let-7b-5p. [file Image_1.TIFF]
